# Spider Webs, Soil or Leaf Swabs to Detect Environmental DNA From Terrestrial Vertebrates: What Is the Best Substrate?

**DOI:** 10.1111/1755-0998.70037

**Published:** 2025-09-04

**Authors:** Aloïs Berard, Julien Pradel, Nathalie Charbonnel, Maxime Galan

**Affiliations:** ^1^ CBGP, INRAE, CIRAD, IRD, Institut Agro Univ Montpellier Montpellier France

**Keywords:** airborne, biodiversity monitoring, environmental DNA, metabarcoding, terrestrial, wildlife

## Abstract

As human activities drive biodiversity decline, effective biomonitoring is more crucial than ever to track species distribution changes and inform conservation and restoration actions. Environmental DNA (eDNA) metabarcoding has emerged as a promising tool for the simultaneous detection of multiple taxa. However, while substrates play a crucial role in eDNA studies, limited research has compared substrate performance for terrestrial vertebrate detection, leaving a critical gap in empirical knowledge for large‐scale application. This study evaluates and compares the effectiveness of three easy‐to‐collect substrates: soil, leaf swabs, and spider webs, for broad terrestrial vertebrate eDNA monitoring. Specifically, we examined taxonomic richness overlaps among substrates, their effects on wild vertebrate detection probabilities, and within‐sample PCR repeatability. We analysed 120 samples from the Landes Forest, an intensively managed temperate forest in Western France, and included additional control samples from the Montpellier zoo to validate our detection capabilities. Using metabarcoding with 12S‐V5 and 16S mam primers, we identified 63 taxa at the genus or species level. Our findings highlight the advantages of substrates that passively accumulate airborne DNA (leaf swabs and spider webs) over soil, and position spider webs as a suitable choice for maximising detection probabilities in rapid eDNA surveys, emphasising their potential for efficient, scalable biomonitoring. Further research is needed to identify factors affecting eDNA detectability from these substrates, aiming to standardise procedures and move from proof‐of‐concept to broad use by researchers and managers.

## 1 Introduction

Human activities are driving a global decline in biodiversity across multiple taxonomic groups, threatening ecosystems and human well‐being (Cardinale et al. 2012; Dirzo et al. 2014). Tackling this crisis requires establishing baseline biodiversity data and continuous monitoring to assess human impacts and fill data gaps that hinder evidence‐based conservation and restoration efforts (Junker et al. 2020; Sutherland et al. 2004). Bridging these gaps necessitates developing efficient and reliable biomonitoring systems that are transferable and applicable for broad, systematic long‐term surveillance.

Traditional biodiversity monitoring techniques face significant limitations for such implementation. These approaches rely on visual surveys or organism trapping followed by morphological identification, resulting in substantial logistical costs, a focus on specific taxa and a reliance on increasingly rare taxonomic expertise (Pearson et al. 2011). Additionally, some methods can be invasive or disruptive, making them unsuitable for studying protected or vulnerable species and raising ethical concerns related to biodiversity monitoring (Lefort et al. 2022).

Emerging non‐invasive and highly scalable technologies offer promising alternatives for biomonitoring. Recently, DNA sequencing of environmental samples has emerged as a fast and effective method for non‐invasively identifying species from DNA traces (Ficetola et al. 2008). Organisms continuously release DNA in the environment through the shedding of bodily fluids and tissues, such as faeces, saliva, and skin cells (Barnes and Turner 2016). Once released, this environmental DNA (eDNA) can persist in various forms, ranging from intracellular states to free DNA which may associate to varying degrees with the surrounding matrix (Nagler et al. 2022). Molecular approaches using eDNA have proven efficient for monitoring biodiversity in hard‐to‐reach locations (Howell et al. 2021) and have enabled detection of invasive, cryptic, and elusive species (Jerde et al. 2011; Koda et al. 2023; Matthias et al. 2021). Its applications range from targeting single taxa of interest using qPCR (Koda et al. 2023) or ddPCR (Capo et al. 2020), to assessing communities through eDNA metabarcoding (Taberlet, Prud'Homme, et al. 2012). By empowering the simultaneous detection of multiple taxa from a single sample, eDNA metabarcoding is particularly promising for large‐scale biomonitoring and broad community assessments (Ruppert et al. 2019; Taberlet, Coissac, et al. 2012).

The eDNA used in monitoring typically corresponds to the total DNA extracted from a specific type of environmental sample (Koziol et al. 2019; Pawlowski et al. 2020), also referred to as a substrate. A wide variety of substrates have been employed across various ecosystems, highlighting both the versatility of this approach and its dependence on the specific environment being studied. For example, in aquatic ecosystems, substrates such as water (Brandt et al. 2021; Koziol et al. 2019), sediments (Brandt et al. 2021; Koziol et al. 2019), biofilms (Rivera et al. 2022) and water‐filtering organisms (Mariani et al. 2019; Weber et al. 2023) are commonly used. Similarly, in terrestrial ecosystems, eDNA has been collected from substrates such as soil (Leempoel et al. 2020), water (Lyet et al. 2021), air (Lynggaard et al. 2024), plant material (Nichols et al. 2012), swabbed surfaces (Newton et al. 2022), snow (Franklin et al. 2019), and artificial materials (Kyle et al. 2022), among others.

Substrate is an essential factor that must be considered in eDNA studies as it directly influences the probability of detection and the diversity of species that can be identified (Koziol et al. 2019). Indeed, the substrate determines the physico‐chemical properties in which the DNA is maintained, which in turn impacts its degradation rate (Collins et al. 2018; Guthrie et al. 2024). In addition, some species interact more frequently with certain substrates than others, due to their behaviour and ecology (Ryan et al. 2022). For example, Brandt et al. (2021) found that deep seafloor sediment was more effective than water for detecting benthic metazoans, while water samples were better for pelagic species, with benthopelagic species detected in both, underscoring the importance of species ecology in substrate selection. Similarly, in a terrestrial context, Allen et al. (2023) found that using roller swabs on tree trunks yielded higher species richness and detection probabilities for arboreal mammals compared to soil sampling.

Recently, the detection of species using airborne eDNA has gained interest and opened up new possibilities for global terrestrial biodiversity monitoring (Bohmann and Lynggaard 2023). Indeed, the ubiquity of air in terrestrial environments, combined with the dispersal properties of airborne particles, suggests that air could serve as an eDNA substrate comparable to water in aquatic ecosystems (Bohmann and Lynggaard 2023), enabling broad and non‐targeted assessments of biodiversity. Airborne DNA has shown promising results in the monitoring of a diverse range of taxa, from bacterial and fungal communities (Gusareva et al. 2019), to plants (Gusareva et al. 2019; Johnson et al. 2021), insects (Roger et al. 2022) and vertebrates (Lynggaard et al. 2024). As with water‐based eDNA sampling (Bessey et al. 2021), airborne DNA can be collected using active or passive methods. Active sampling involves artificially drawing air through a sampler or filter using a powered device, whereas passive sampling relies on the natural deposition of airborne particles onto a surface (Bohmann and Lynggaard 2023). Passive airborne eDNA collection methods could offer several advantages over active sampling approaches, including minimal material and logistical requirements. Many of these methods also avoid the need for extensive installations, allowing multiple samples to be collected during a single field session. This could make them particularly valuable for rapid biodiversity assessments and for surveying remote or hard‐to‐access environments. Moreover, the accessibility of substrates able to passively collect airborne eDNA could make them suitable for large‐scale monitoring systems and opens the door for integration into citizen science programs, further enhancing monitoring scalability and reach (Bohmann et al. 2014; Chandler et al. 2017).

Up to now, most studies using eDNA for terrestrial vertebrate detection remain at the proof‐of‐concept stage. However, wild terrestrial vertebrates (i.e., Tetrapoda) are among the taxonomic groups most severely affected by anthropogenic pressures, with widespread population declines and an alarming number of species facing extinction (Ceballos et al. 2017; WWF 2024). Although species distribution data have improved over the years, many regions, especially remote or inaccessible areas, remain poorly surveyed (Moura and Jetz 2021). Furthermore, the dynamic nature of species distributions due to environmental changes, habitat loss, and climate change further complicates our understanding of where wild vertebrates are located at any given time. Therefore, given their crucial role in maintaining ecosystem functionality and their importance to human well‐being (Ceballos et al. 2020; Keesing et al. 2010), this group urgently requires effective biomonitoring to guide conservation efforts.

While a wide range of substrates has been explored to detect terrestrial vertebrates (reviewed by Newton, Allentoft, Bateman, van der Heyde et al. 2025) standardised comparative studies are needed to evaluate their differences in performance for taxa‐specific DNA detection. Such studies are crucial for optimising substrate selection in terrestrial contexts and improving eDNA‐based monitoring systems for wild vertebrates. However, comparisons between multiple substrates remain scarce and existing studies highlight substantial variability in species diversity and detection rates (Allen et al. 2023; Kyle et al. 2022; Newton et al. 2022; van der Heyde et al. 2020).

In this study, we aim to address this challenge by confirming the ability of three substrates to detect vertebrate DNA and comparing their performance as promising candidates for broad terrestrial vertebrate monitoring. These substrates are widely distributed across terrestrial ecosystems, require minimal resources, and are well‐suited for rapid, single‐step sampling. The first substrate is soil, one of the most commonly used substrates for terrestrial vertebrate detection, second only to water which is not suitable for every terrestrial ecosystem (Newton, Allentoft, Bateman, van der Heyde et al. 2025). The other two substrates are relatively new to this application, having only recently been explored for vertebrate detection. These substrates likely rely primarily on the passive accumulation of DNA from airborne particles through settling or entrapment. The first of these is leaf swab, which successfully permitted detection of vertebrate DNA in the tropical rainforest of Kibale National Park, Uganda (Lynggaard et al. 2023). The second is spider web, which enabled the detection of vertebrates in both the Perth Zoological Park and the Karakamia Wildlife Sanctuary in Australia, under a mediterranean climate (Newton et al. 2024).

In order to evaluate and compare these substrates, we conducted our study in the Landes forest, a vast temperate forest characterised by an oceanic climate, sandy soils, and vast maritime pine ( *Pinus pinaster* ) plantations. While small remnants of broadleaved forests persist, the dominance of pine monocultures creates ecological challenges. Tree diversification initiatives are currently being explored to enhance biodiversity and mitigate these risks. In this context, efficient eDNA biomonitoring could offer a valuable tool to evaluate the short and long‐term impacts of these programmes on biodiversity and provide essential data for conservation and management.

More specifically, our objectives were (i) to assess whether soil, leaf swabs, and spider webs can accumulate detectable wild vertebrate eDNA in both wild and controlled environments and to assess their potential for monitoring biodiversity in the specific context of the Landes forest; (ii) to compare the substrates in terms of taxonomic richness, taxonomic‐specific detection probabilities, and within‐sample PCR repeatability, in order to identify the best substrate(s) and provide valuable insights for their potential use in long‐term biodiversity monitoring.

## 2 Material and Methods

### 2.1 Study Sites

**FIGURE 1:**
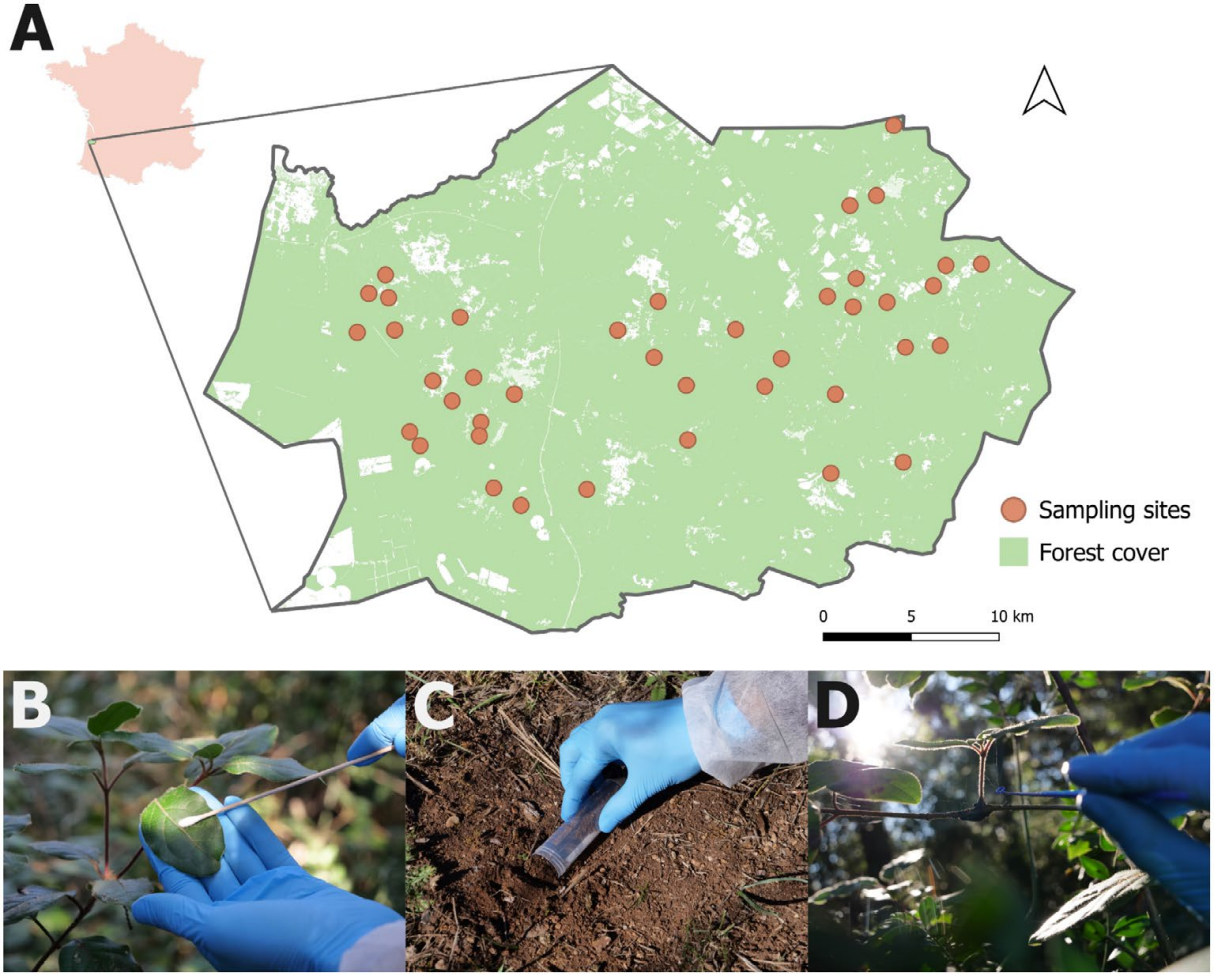
(A) Map of sampling sites within the Landes Forest (France). Forest cover is the combination of coniferous and broadleaved forest cover data produced by CNES for the Theia data centre (www.theia‐land.fr) using Copernicus products. The map was created using QGIS (v3.40.2). (B) Leaf sampling procedure. (C) Soil sampling procedure. (D) Spider web sampling procedure.

Sampling was performed in June 2024 within a 1050 km^2^ area of the Landes Forest in southwestern France (44°33′38.2”N 0°46′36.7”W; Figure 1A). It was conducted along 40 sites, with many overlapping those previously surveyed by Plat et al. (2024). Additionally, a positive control site for which the captive vertebrate fauna is known was included (Zoological park in Montpellier) solely to verify the ability of our protocols to detect locally present species. Sampling at this site was conducted near the enclosure of two giant anteaters ( *Myrmecophaga tridactyla* ) within a tropical greenhouse (Figure S1) that also housed other Neotropical species such as Grey‐winged Trumpeters ( *Psophia crepitans* ) and Brazilian porcupines ( *Coendou prehensilis* ), alongside several synanthropic species commonly found in urban environments (e.g., black rats: *Rattus rattus* ).

### 2.2 Sample Collection

For each of the sampling sites, three types of substrate were collected (Figure 1), with one sample per substrate type. First, for each site, 50 mL of topsoil were collected at 10 points, spaced 5 m apart. They were pooled in a resealable plastic bag where the final 500 mL were homogenised by kneading and shaking the bag for 3 min. Then a 50 mL sample was stored in a Falcon tube. Second, leaf swabs were taken for each site by rubbing a sterile cotton swab over leaves from a broadleaved woody plant, primarily pedunculate oak ( *Quercus robur* ) or alder buckthorn ( *Frangula alnus* ). One to three leaves were swabbed for approximately 1 min in order to cover a surface area of about 100 cm^2^. Finally, two to three spider webs were collected from each site using the same sterile plastic inoculation loop and placed together in a single 2 mL tube, forming a single pooled sample. Both the collected spider webs and leaves used for swabbing were located within a similar height range, from ankle to chest level. To minimise operator bias, all samples were collected by the same person. They were kept dry and stored at −20°C within 8 h of collection.

### 2.3 eDNA Extraction

Molecular biology procedures were conducted in laboratories organised with strict separation of activities, particularly pre‐ and post‐PCR. To prevent contamination with previously extracted vertebrate DNA, all DNA extraction steps were performed in a laboratory dedicated to plant studies and free from PCR‐amplified vertebrate DNA. Operators were equipped with gowns, gloves, and surgical masks. Surfaces were decontaminated using DNA Away (Thermo Scientific) and all equipment used was sterile and single‐use. Negative DNA extraction controls (*n* = 3) were included in each extraction session, with each session dedicated to a single substrate type. These controls were processed alongside the samples until sequencing.

Soil samples were extracted following Taberlet, Prud'Homme, et al. (2012). Briefly, 10 g of each soil sample was taken and added to 20 mL of saturated phosphate buffer (Na2HPO4; 0.12 m; pH ≈8). This solution was homogenised by inverting for 15 min, then 1 mL of supernatant was transferred to a 2 mL tube and centrifuged at 10,000 g for 10 min. 400 μL of clear supernatant was extracted using the NucleoSpin Soil Kit (Macherey Nagel), omitting the lysis step and following the manufacturer's instructions.

Leaf swabs were extracted using the QIAamp DNA Investigator Kit (Qiagen) following the manufacturer's instructions except for elution which included an incubation step of 37°C for 15 min after adding the ATE buffer with 0.05% Tween‐20 as recommended by Lynggaard et al. (2023).

Spider webs were extracted using the DNeasy Blood and Tissue Kit (Qiagen) according to Newton, Allentoft, Bateman, Campbell, et al. (2025) recommendations. Digestion volumes were tripled (60 μL proteinase K+540 μL ATL buffer), and lysis volumes were doubled (400 μL digestion +400 μL AL buffer +400 μL absolute ethanol).

### 2.4 eDNA Amplification and Sequencing

As proposed by Lynggaard et al. (2022) and Newton et al. (2024), we used the universal PCR primers amplifying an approximately 100 bp fragment of the V5 variable region of the 12S rRNA gene from vertebrates (12S‐V5‐F: 5‘‐TAGAACAGGCTCCTCTAG‐3’; 12S‐V5‐R: 5‘‐TTAGATACCCCACTATGC‐3’) (Riaz et al. 2011) and an approximately 96 bp fragment of the 16S mitochondrial gene from mammalians (16Smam1‐F: 5‘‐CGGTTGGGGTGACCTCGGA‐3’; 16Smam2‐R: 5‘‐GCTGTTATCCCTAGGGTAACT‐3’) (Taylor 1996). Each DNA sample was sequenced in three independent PCR replicates for each gene, with the addition of negative PCR and indexing controls to check for possible contamination. Amplicon libraries were prepared by 2‐step PCR as described in Galan et al. (2018) and using Unique Dual Indexes (UDIs) to avoid sequence misassignment phenomena. PCR products were checked on agarose gel, then pooled for each substrate and gene, and normalised after quantification by qPCR using the KAPA Library Quantification kit (Roche), following the manufacturer's instructions. Sequencing was performed in pair‐end 2 × 150bp on MiSeq (Illumina) using a 300v2 cartridge. Full procedure details are available in Data S1.1.

### 2.5 Sequence Analyses and Taxonomic Assignment

Sequences were analysed using the FROGS pipeline (Find, Rapidly, OTUs with Galaxy Solution; Escudié et al. 2018) with the Swarm clustering method (Mahé et al. 2021) employing the *d = 1* parameter in order to generate Amplicon Sequencing Variants (ASVs). ASVs with fewer than two reads or those absent in at least two different libraries (including technical replicates) were excluded. ASVs with read counts equal to or below those of the substrate's negative control were considered null. Remaining ASVs were matched against the GenBank core nucleotide database for taxonomic assignments using BLASTN. Percentages of identity greater than 98% were considered sufficient for assignment to the species level when a single species gave the best match. When multiple assignments shared equivalent best matches, we assigned the ASV to the common taxonomic rank unless only one of the matching species was known to occur within the study area, in which case that species was retained. Finally, ASVs affiliated to the same taxa were grouped together and unassigned ASVs were discarded. For each marker, the technical replicates were then combined by summing reads per sample. A sample was considered positive for a taxa if it contained corresponding reads. Full procedure details are available in Data S1.2. Taxonomic assignments obtained for 12S‐V5 and 16Smam regions were compared and integrated to keep the best taxonomic resolution (i.e., up to species level) possible for each taxa detected (see Table S1). Data from both genes were then combined by summing reads for each sample.

### 2.6 Statistical Analysis

All data exploration and statistical tests were performed with R (v4.4.1) (R Core Team 2024). The Euler plot was created with eulerr (v7.0.2) (Larsson 2024). Wild vertebrate accumulation curves for each substrate were drawn using the iNEXT package (v3.01) (Hsieh et al. 2016). The cladogram was constructed based on systematic literature (Jones et al. 1984; Stiller et al. 2024) and annotated using iTOL (v7.0) (Letunic and Bork 2021). Statistical modelling was performed exclusively on samples from the Landes Forest.

We tested the effect of substrate type (i.e., soil, leaf swab or spider webs) and vertebrate taxonomic class (i.e., Aves or Mammalia) on the probability of detecting wild vertebrate eDNA (i.e., excluding domestic vertebrates and humans). We applied a generalised linear mixed‐effects model (GLMM) using a logistic regression. The response variable was the detection probability of each taxon per sample (vertebrate detection event = 1; no detection = 0). The full model included substrate type, vertebrate class, and their interaction as fixed effects, with sample and taxa identity included as random factors. Model selection was performed on full models using the corrected Akaike Information Criterion (AICc). Pairwise *post hoc* comparisons were conducted on estimated marginal means using Tukey's range test (α = 0.05).

Additionally, within‐sample detection repeatability was modelled using a similar framework, excluding the inclusion of taxonomic class. Each detection event was classified as 0 or 1, respectively, if less than or more than half of the PCR replicates were positive, with the sample included as a random factor.

Models were generated, selected, and controlled using lme4 (v1.1–35.5) (Bates et al. 2015), MuMIn (v1.48.4) (Bartoń 2025), DHARMA (v0.4.6) (Hartig et al. 2024) and emmeans (v1.10.3) (Lenth 2025).

## 3 Results

### 3.1 Sampling

A total of 123 samples, representing one sample per substrate type at each of the 41 sites, were analysed in triplicate for each DNA marker targeting vertebrate eDNA. This was done alongside three extraction controls, three PCR controls, and three indexing controls for both markers, resulting in a total of 756 amplicon libraries in a single sequencing run.

### 3.2 Sequence Analyses and Control

A total of 3,136,792 reads were generated across the two markers. Initial read counts were consistent across markers and substrates ranging from 529,039 to 616,102, except for 12S‐V5‐soil which had slightly fewer reads (315,712). Soil samples exhibited the highest proportion of read loss during the denoising and dereplication steps, with 45% for 12S‐V5 and 40% for 16Smam. Other substrates showed notably lower losses, ranging from 0.3% to 6.7%, as observed in leaf swabs, for 16Smam and 12S‐V5 respectively. After all filtering steps, 1,303,285 reads remained for 12S‐V5 and 1,528,798 for 16Smam, with human ASVs accounting for 69% and 75% of the reads, respectively. For the 12S‐V5 marker, 11 spider web samples and 11 soil samples had fewer than 1000 total reads. For the 16Smam marker, 12 spider web samples and one soil sample fell below those 1000 reads. These samples with less than 1000 reads can be attributed to weak or failed PCR reactions observed on agarose gel. No leaf swab samples exhibited such low read counts (ranging from 4427 to 22,649 reads). Details of sequence analyses are provided in Table S2.

Contamination was detected in eight amplicon library replicates. For the 12S‐V5 marker, one PCR replicate from the negative control of soil DNA extraction contained 1463 reads of pig ( *Sus scrofa* ), while two PCR replicates from the leaf swab samples showed contamination with sardine ( *Sardina pilchardus* ). For 16S mam, one PCR replicate from the negative control of leaf swab DNA extraction contained 1392 reads of black rat ( *Rattus rattus* ). Additionally, sardine contamination was detected in one negative control from the spider web DNA extraction with both markers.

Contamination by sardines was linked to their use as bait in a simultaneous study carried out on the same sites. Human and sardine occurrences were not further considered, as their presence was attributed to contamination during the sampling or DNA extraction process. Rats and pig occurrences were disregarded when their read counts fell below those observed in the substrate extraction controls.

Control samples collected from the zoo allowed the detection of locally present fauna by yielding 12 non‐human vertebrate taxa using the 12S‐V5 marker and eight using the 16Smam marker. Notably, the anteaters were detected across all substrate types with 12S‐V5 but were not detected with 16Smam.

### 3.3 Taxonomic Affiliation

Considering all samples, including those from the control site at Montpellier Zoo, the 12S‐V5 marker enabled the identification of 66 distinct non‐human vertebrate taxa: 49 identified at the species level, six at the genus level, eight at the family level, and three at the order level. These comprised 38 birds (Aves), 22 mammals (Mammalia), three reptiles (Reptilia), and three amphibians (Amphibia). The 16Smam marker enabled the identification of 49 taxa: 40 at the species level, seven at the genus level, one at the family level, and one at the order level, including 24 birds, 23 mammals, and two amphibians. Non‐human vertebrate detections occurred in 87 samples overall: 73 with the 12S‐V5 marker and 77 with the 16Smam marker.

**FIGURE 2:**
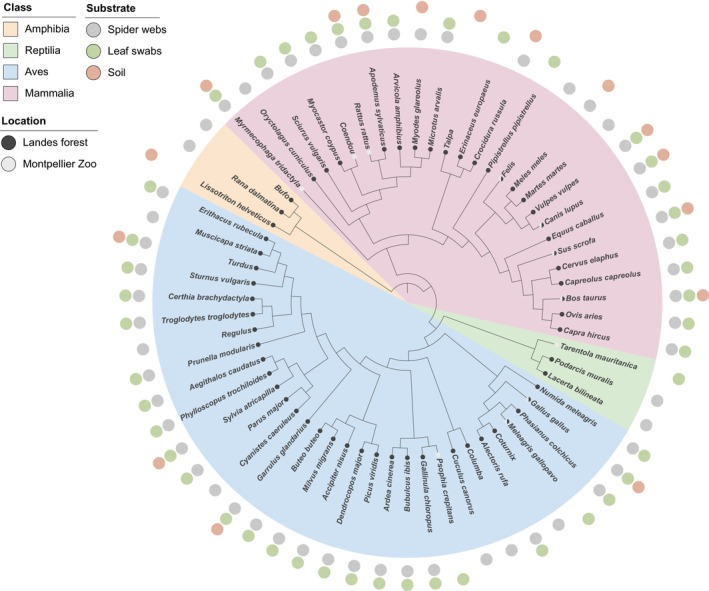
Terrestrial vertebrate taxa identified below the family level from soil, leaf swab, and spider web samples collected across 40 sites in the Landes forest and one control site at Montpellier Zoo, using 12S and 16S rRNA gene markers. Taxonomic assignments represent the homogenised results obtained from both markers. Each site was represented by one sample per substrate. Humans and *Sardina pilchardus*, identified as contaminants, were not represented.

After taxonomic integration between markers, we obtained a final list of 73 non‐human taxa. These taxa were identified at different taxonomic ranks: 55 at the species level, eight at the genus level (Figure 2), seven at the family level, and three at the order level (Table S3).

For all subsequent results, only samples collected in the Landes forest were considered, resulting in a total of 67 taxa with 51 at the species level, seven at the genus level, six at the family level, and three at the order level. Several detected taxa were domestic or human‐associated vertebrates, including pigs ( *Sus scrofa* ) in 48 samples, chicken ( *Gallus gallus* ) in 39, or cows ( *Bos taurus* ) in 23. As the focus of this study was wild vertebrates, domestic species such as pig, chicken, cow, goat ( *Capra hircus* ), sheep ( *Ovis aries* ), dog ( *Canis lupus* ), horse ( *Equus caballus* ), turkey ( *Meleagris gallopavo* ), and rabbit ( *Oryctolagus cuniculus* ) were not considered in further analyses. Although Ring‐necked Pheasant ( *Phasianus colchicus* ), Helmeted Guineafowl ( *Numida meleagris* ), and Red‐legged Partridge ( *Alectoris rufa* ) can be bred by humans, they were not classified as domestics, as their presence in the forest was likely due to free‐ranging individuals. Taxa that could not be assigned to the genus or species level were further excluded as a precaution (*n* = 9), leaving 49 taxa in the final dataset.

In this final dataset, birds were the most diversified class of wild vertebrates with 28 taxa detected, followed by mammals with 16 taxa, amphibians with three taxa and reptiles with two taxa. With 140 detection events, birds were also the most often detected, followed by mammals with 62 events, amphibians with four and reptiles with three. With 14 positive samples, the Common Chiffchaff ( *Phylloscopus collybita* ) was the most detected wild taxon, while the most detected mammals were the European wood mouse ( *Apodemus sylvaticus* ) and the greater white‐toothed shrew ( *Crocidura russula* ) with both nine positive samples.

**FIGURE 3:**
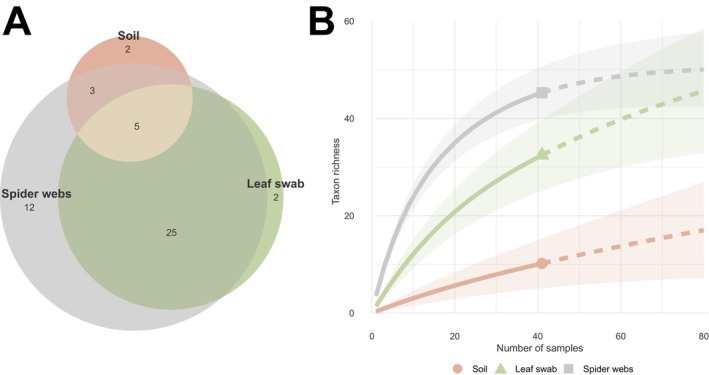
(A) Euler plot showing shared detection of wild vertebrate taxa (i.e., excluding human‐associated taxa) among substrates (soil, leaf swab, and spider webs) collected within the Landes forest. (B) Rarefaction curves (mean ± SE) for wild vertebrate taxa detected per substrate. Solid lines represent observed richness, while dotted lines indicate extrapolated richness. Detection results are based on the combined analyses of 12S and 16S gene regions, retaining only taxa identified below the family level. Each substrate was represented by 40 samples.

Amphibian taxa were detected exclusively in spider webs (*n* = 2) and soil samples (*n* = 1), whereas reptile taxa (*n* = 2) were detected only with spider webs. Spider web samples enabled the detection of 45 distinct taxa with an average of 3.5 taxa per sample in the positive samples, which was higher than leaf swabs with 32 taxa (1.4 taxa per sample) and soil with 10 taxa (0.3 taxa per sample) in terms of taxonomic richness (Figure 3A). Species accumulation curves comparing substrates show that taxon richness increases more rapidly for spider webs than for other substrates (Figure 3B). Each curve shows an increasing trend, with no plateau yet reached after 40 samples assessed, while the soil curve shows no sign of inflexions, indicating slower taxon accumulation.

### 3.4 Statistical Analyses

#### 3.4.1 Detection Probability for Wild Vertebrate Taxa

**FIGURE 4:**
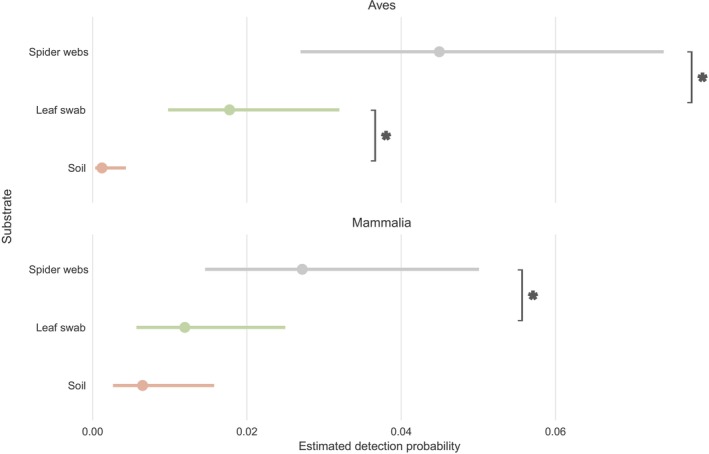
Estimated marginal means of detection probability of vertebrate detection per substrate and taxa class. Bars represent 95% CI. Asterisks indicate significant differences based on a Tukey test (α = 0.05). Results are based on wild vertebrate taxa only (i.e., excluding human‐associated species), detected in the Landes forest, and do not include amphibians or reptiles. Each substrate is represented by 40 samples. Spider web samples consist of pools of 2 to 3 webs. For leaf swabs, approximately 100 cm^2^ of leaf surface was swabbed during 1 min per sample. Soil samples consist of 10 g subsamples taken from an initial 500 mL soil sample afterhomogenisationn.

Amphibian and reptile taxa were not considered in the dataset used to model detection probability as those classes were not sufficiently represented. Domestic taxa and humans were also excluded from this dataset. The best model selected included all fixed effects (GLMM: AICc = 1431, df = 8, log‐likelihood = −707). It showed that vertebrate detection probability was influenced both by substrate and the interaction between substrate and the class of related vertebrates (Figure 4). For birds, leaf swabs had significantly lower detection probability than spider webs (*Z* = −2.673; *p‐value* = 0.02) but higher than soil (Z = 3.970; *p‐value* < 0.001). For mammals, the only significant probability difference was observed between soil and spider web samples (*Z* = −2.933; *p‐value* < 0.001). Overall, spider webs surpassed other substrates in detection probability. They yielded 140 detection events, compared to 56 events for leaf swabs, and 13 for soil.

#### 3.4.2 Repeatability of Taxa Detection Among Replicates

Detection repeatability among replicates was modelled using a similar framework, but without including taxonomic class as a factor. This allowed us to retain taxa from all vertebrate classes, including Amphibia and Reptilia. The best model selected to explain the repeatability of vertebrate detection included the substrate (GLMM: AICc = 273, df = 4, log‐likelihood = −132). It only showed a significant difference between leaf swabs and spider webs (*Z* = −2.897; *p‐value* = 0.01; Figure S2). The confidence interval for soil's estimated probability (95% CI = [0.038; 0.45], *n* = 13) was 2.4 times wider than that for spider webs and 1.9 times wider than that for leaf swabs. Among the substrates, spider webs demonstrated the highest within‐sample repeatability, with 46% of detection events replicated in more than half of the PCR replicates (leaf swabs: 23%; soil: 15%; Table S4).

## 4 Discussion

### 4.1 Monitoring Terrestrial Vertebrate Diversity From eDNA

In this study, we compared the effectiveness of soil, leaf swab and spider webs as eDNA substrates for surveying global terrestrial vertebrate diversity through metabarcoding in a French temperate forest. All three substrates successfully detected wild vertebrates, but spider webs showed comparatively higher performance in terms of taxonomic richness, detection probability, and within‐sample PCR repeatability. Leaf swabs ranked as the second most effective substrate, while soil samples provided fewer detections and diversity. With a substantial taxonomic overlap among substrates, our findings highlight the potential advantages and limitations of each one as valuable fast alternatives to other eDNA collection approaches.

With more than 50 taxa identified at the genus or the species level, our eDNA survey revealed a broad spectrum of the area's vertebrate biodiversity, including the invasive nutria ( *Myocastor coypus* ) and the protected palmate newt ( *Lissotriton helveticus* ). Consistent with the high level of anthropisation in our study area, a substantial proportion of detections were linked to domestic taxa. Most vertebrate detections were of birds and mammals, while reptiles and amphibians were detected less frequently across all substrates. The lower detection rates for these groups may reflect smaller biomass compared to birds and mammals in the Landes forest. For reptiles, this pattern could also be explained by the “shedding hypothesis” which suggests that organisms with hard exterior (e.g., arthropods and reptiles) may shed eDNA at lower rates than those with soft ones (Adams et al. 2019; Nordstrom et al. 2022). This limited shedding could explain the low detectability of reptiles‐derived sequences observed in some eDNA studies (Lacoursière‐Roussel et al. 2016; Newton et al. 2024). Amphibians, on the other hand, lead semi‐aquatic lives, dividing their time between land and water. Their dependence on aquatic habitats means that some of our sampling sites may not sustain amphibian populations. Furthermore, their semi‐permeable skin may imply shedding rates that differ from those of birds and mammals. While their shedding mechanism poses few challenges in aquatic eDNA studies (Sun et al. 2024), it could complicate detection in terrestrial environments. These ecological and physiological characteristics emphasise the importance of considering the behaviour and lifestyle traits of target species for substrate selection and sampling strategy in eDNA studies. Targeted eDNA methods designed for reptiles, such as those employed by Matthias et al. (2021) and Kyle et al. (2022), have yielded promising results by focusing on microhabitats under cover objects, where snake DNA is more likely to accumulate due to their thermoregulatory behaviour. For amphibians, aquatic substrates likely remain the most effective given their ecological dependence on water and shedding processes (e.g., gamete release and larval hatching; Buxton et al. 2017; Sun et al. 2024). Non‐targeted methods, such as spider webs and leaf swabs used in our study, appear well‐suited for monitoring most birds and mammals, which shed DNA consistently into the environment through particles such as hairs, feathers, and skin fragments. However, for species with low DNA shedding or unique ecological traits, targeted sampling strategies based on species' ecology and substrate availability may improve detection. Overall, combining non‐targeted with complementary targeted sampling strategies would provide a more comprehensive assessment of terrestrial vertebrate communities.

### 4.2 Methodological Factors and Technical Challenges

#### 4.2.1 Number of Replicates

Discrepancies observed between PCR replicates reaffirm replication as a critical parameter to consider in order to limit false negatives in eDNA studies, as previously discussed (Ficetola et al. 2015). The differences in repeatability underscore the importance of considering this parameter during substrate selection. Properly accounting for repeatability could help reduce both costs and false negatives.

#### 4.2.2 Markers and Database

The combined use of the 12S‐V5 and 16Smam regions enabled the detection of a greater number of vertebrate species, consistent with previous studies (Lynggaard et al. 2024; Newton et al. 2024). While both markers detected a comparable number of mammalian taxa, the 12S‐V5 primers allowed for the detection of substantially more non‐mammalian vertebrates, including more than a dozen of additional bird species and all reptile detections. This result is unsurprising as 12S‐V5 primers were designed as a general vertebrate marker (Riaz et al. 2011), whereas 16Smam was specifically developed for mammals (Taylor 1996) and likely lacks sufficient taxonomic coverage for other vertebrate classes (Ficetola et al. 2010). Additionally, a *post hoc* analysis revealed critical mismatches between the sequences of the 16Smam1 forward primer (10 bases from the 3′ end), the 16Smam2 reverse primer (nine bases from the 3′ end) and the 16S anteater sequence, while no mismatch was observed between the 12S‐V5 anteater sequence and the corresponding primers. These mismatches likely explain the failure to detect zoo anteaters using the 16S rRNA gene, despite other zoo species being successfully identified with this marker. Critical mismatches in the 16Smam primers, which hinder PCR amplification of certain mammalian groups, have recently been reported by Campbell et al. (2024).

Metabarcoding is inherently reliant on sequence databases, which can vary in completeness between markers. This database dependency is a fundamental challenge for eDNA monitoring, particularly for understudied taxa, as observed in certain studies conducted in neotropical regions (Sales et al. 2020). Due to multiple assignments, some of our sequences were identified above the species level. This may result from gaps in databases or insufficient resolution of the amplified markers for certain species. Using longer amplicons could overcome these limitations, but this approach may reduce the probability of detection, as eDNA is often fragmented in short sequences due to degradation.

#### 4.2.3 Contaminations

Despite following rigorous sampling and laboratory protocols, some contaminants were detected in our metabarcoding data. Human DNA, commonly found in eDNA studies (Leempoel et al. 2020; Newton et al. 2024), even when using specific blockers (Frere et al. 2024; Lynggaard et al. 2024), was identified. Rare contaminations from black rats and sardines were observed and accounted for. Pig DNA was also found in one extraction control sample which is unsurprising as some domestic animals are known laboratory contaminants (Leonard et al. 2007). However, some of the pig DNA may also originate from wild boars, which are known to inhabit the study area. To ensure confidence in our comparative analyses, we applied conservative and stringent criteria, including the exclusion of domestic species. Nonetheless, these results underscore the sensitivity of eDNA methods and highlight the risk of erroneous biodiversity surveys if false positives are not carefully managed (Beng and Corlett 2020).

### 4.3 Limits and Advantages of Soil Samples

Soil samples performed significantly worse than leaf swabs and spider webs in surveying terrestrial vertebrates. This finding aligns with previous multi‐substrate studies highlighting the lower effectiveness of soil eDNA for broad biodiversity assessments (van der Heyde et al. 2020; Cowgill et al. 2025), and for vertebrate detection in particular (Allen et al. 2023; Matthias et al. 2021; Newton et al. 2022; Ryan et al. 2022), relative to alternative substrates. The limitations of soil as a substrate can be partly attributed to its high local heterogeneity, which is often addressed by collecting larger volumes of soil (Taberlet, Prud'Homme, et al. 2012). However, intrinsic soil properties (e.g., pH, UV exposure, temperature, humidity and structure) and content (e.g., cations, microbial communities and enzymes) are known to influence DNA degradation or inhibit downstream processes like DNA extraction and PCR amplification (Andersen et al. 2012; Guthrie et al. 2024; Sirois and Buckley 2019; Young et al. 2014). For example, the highly acidic soils in our study area (Augusto et al. 2006) may have accelerated DNA degradation, as low pH is known to promote DNA hydrolysis (Seymour et al. 2018). To optimise the use of soil eDNA, additional cleaning steps can be employed to remove components that hinder detection rates, such as PCR inhibitors (e.g., humic acid; Tsai and Olson 1992) which probably contributed to the PCR failures observed for the soil samples in our study. However, these steps can lead to reduced DNA concentrations (McKee et al. 2015) and may still fail to achieve detection efficiencies comparable to those of other substrates (Newton et al. 2022). Consequently, vertebrate species detection using soil would require more extensive sampling, increased replication, and more labor‐intensive methods to process sufficient material and mitigate the effects of inhibitors.

Despite these challenges, soil remains one of the most ubiquitous and accessible substrates, making it a practical choice in certain contexts. Numerous studies have successfully used soil eDNA for biodiversity surveys, notably for mammal and bird communities (Leempoel et al. 2020; Tetzlaff et al. 2024). Furthermore, soil is indispensable to study the specific below‐ground communities (Bienert et al. 2012; Rosa et al. 2020). In our study, we observed a high proportion of reads eliminated during the first bioinformatics steps for soil samples. These excluded reads predominantly correspond to non‐target sequences, such as those from invertebrates (e.g., Annelida) and microorganisms, often falling outside the expected amplicon size ranges. Additionally one of the few vertebrates found uniquely in soil were moles (*Talpa* sp.), which exhibit a subterranean lifestyle. This highlights that while soil may generally perform poorly for detecting vertebrate‐derived sequences, it could be effective for species whose behaviour or ecology involves frequent interaction with soil and should therefore not be overlooked as a substrate.

While detection probabilities for mammals were similar between soil and leaf swabs, significant differences were observed for birds. This may reflect that although some airborne DNA likely settles onto soil, its detectability is limited by low concentrations, rapid degradation, and the difficulty of amplifying DNA from this substrate. As a result, eDNA detection from soil may depend more heavily on direct biological contact, which can lead to higher local DNA concentrations, compared to other substrates. Consequently, soil may be less effective at capturing DNA from bird species with limited ground‐foraging behaviour but more suited to detect ground‐dwelling or burrowing organisms that interact closely with it, such as the mammals present in our study area. Altogether, our observations seem to support the idea that while soil is well suited for metabarcoding studies targeting subterranean fauna, invertebrate macrofauna, and microbial communities, it tends to be less effective for detecting above‐ground vertebrate eDNA in adequate quality and quantity. For broad, non‐targeted surveys of terrestrial vertebrates, we recommend prioritising alternative substrates like spider webs or leaf swabs, which have demonstrated superior detection performance for most vertebrates.

### 4.4 Comparison of Leaf Swabs and Spider Webs as eDNA Substrates

Swabbing offers a non‐disruptive method of sample collection, in contrast to spider web harvesting, which involves removing a structure that is energetically costly for orb‐weaver spiders to build and often critical for their foraging or shelter (Blackledge et al. 2011). However, despite showing some promise, leaf swabbing was significantly less effective than spider webs and presented practical challenges during our study. At certain sites, plant communities were dominated by thorny species, providing few plants with leaves suitable for swabbing. This limitation might reduce the applicability of leaf swabs in such environments compared to the tropical forest where this substrate was initially tested (Lynggaard et al. 2023). In cold regions, seasonal leaf loss in many temperate species may further restrict leaf availability, potentially limiting the method's applicability for year‐round monitoring. In contrast, even though spider activity declines at low temperatures (Wolfgang 2011), some webs might persist through winter. They may also occur in a broader range of environments including monospecific conifer plantations and the interiors of buildings or caves.

The differences in performance observed between leaf swabs and spider webs may stem from several factors. Notably, while spider web collection does not depend on the absorption capacity of an external material, the effectiveness of swabs as eDNA substrates may be limited by the properties of the swab material itself, since some materials are known to influence DNA collection and extraction efficiency (Verdon et al. 2014).

Performance differences may also result from variations in eDNA accumulation properties, particularly when substrates persist in the environment over time, such as certain webs and leaves. Differences in the detection of species that are temporarily present, such as migratory or hibernating species, can highlight distinct accumulation properties of substrates. However, in our study, the detection of these species is more likely to reflect their current occupancy rather than eDNA accumulation. Although some of the detected bird species, such as the Spotted Flycatcher ( *Muscicapa striata* ) are known to migrate, all migratory birds are typically present in France by July, the month in which we collected our samples.

Differences in sampling design likely also contributed to the observed disparity in performance. In our study, leaf swabbing involved a limited leaf surface (i.e., 100cm^2^), while two to three spider webs were pooled without surface limit, potentially resulting in a higher quantity of DNA in web samples. Refining the leaf swabbing protocol, for example, by increasing the swabbed surface or by swabbing multiple leaves within an extended time period, as proposed by Lynggaard et al. (2023), may help enhance DNA yield and improve detection rates.

It is also important to note that swabbing is not confined to leaves. Previous research has demonstrated the successful recovery of eDNA from other surfaces such as tree trunks (Allen et al. 2023; Newton et al. 2022), as well as from artificial materials (Kyle et al. 2022; Matthias et al. 2021). Expanding swabbing to include a wider range of surfaces could improve DNA collection.

### 4.5 Promises of Spider Webs for eDNA Monitoring and Progress Still to Be Achieved

Spider webs have only recently emerged as a highly promising eDNA substrate, with few proof‐of‐concept studies conducted so far. Recent DNA metabarcoding studies have shown their ability to capture DNA from spiders, their prey, and associated fungal and bacterial communities (Gregorič et al. 2022; Xu et al. 2015). More recently, spider webs have also been used to study terrestrial vertebrate eDNA (Newton et al. 2024). As the second study to use spider web eDNA for vertebrate detection, our research confirms its effectiveness in detecting these taxa. Moreover, as the first one to compare spider webs to other substrates, we highlight their seemingly higher performance. Their great apparent performance may stem from their intrinsic properties. For example, their adhesive nature and their three‐dimensional structure may offer an advantage in trapping airborne DNA or vertebrate tissues like hairs. Additionally spider webs' ability to trap insects could provide an extra source of vertebrate DNA, as invertebrate‐derived DNA (iDNA) from sarcophagous, hematophagous or coprophagous arthropods caught in the web may contribute to vertebrate detection (Calvignac‐Spencer et al. 2013; Massey et al. 2022).

Although spider webs performed significantly better than soil and leaf swabs, a subset of samples yielded very low quantities of sequencing reads, due to weak or failed PCR amplifications. Most of these problematic samples exhibited a brownish appearance after extraction, unlike the others. This suggests that the failures may have been caused by the presence of inhibitors, likely due to an excessive amount of starting material, particularly debris such as plant fragments, dust particles, and insects caught in the webs. To optimise the method, future collections could prioritise cleaner substrates with minimal debris, reduce the amount of material collected, or use DNA extraction kits designed to remove inhibitors.

While external factors such as precipitation are known to influence eDNA detection probability (Johnson et al. 2023) and should be accounted for in downstream analyses (e.g., species distribution models), it is also important to identify spider web‐specific features that impact eDNA collection. Optimising these factors and standardising protocols would enhance the reliability of comparisons between studies and strengthen efforts to align eDNA surveys with traditional biodiversity monitoring methods. Parameters like sampling height and spider species should be evaluated, as they may affect spider webs' effectiveness as eDNA substrates. Indeed, the height of the spider webs is likely to influence the proximity of webs to specific species and microhabitats. Moreover, webs can vary significantly in architecture, suspension duration, composition depending on spider species (Eberhard 2020), potentially affecting their efficacy in eDNA collection.

## 5 Conclusion

Our study reasserts eDNA as a promising tool for terrestrial biodiversity monitoring, offering new opportunities for researchers and managers to assess species distribution and track conservation or restoration targets. We demonstrated in natural settings that leaf swabs and spider webs outperformed soil samples, making them particularly promising for non‐targeted vertebrate surveys. Spider webs, in particular, stood out as the most effective substrate for taxonomic richness, detection probability, and PCR repeatability under our tailored sampling protocols, which were designed separately for each substrate. Combined with their minimal sampling effort, these advantages make spider webs a strong candidate for future large‐scale vertebrate monitoring programmes. Further research is yet needed to optimise this method for practical application in next‐generation biodiversity monitoring, moving beyond proof‐of‐concept to reliable and scalable solutions.

## Author Contributions

A.B. Conceptualization, Formal analysis, Investigation, Data Curation, Writing, Original Draft, Visualisation, Writing, Review and Editing. J.P. Investigation, Resources, Data curation. N.C. Conceptualization, Validation, Writing, Review and Editing, Supervision, Project administration, Funding acquisition. M.G. Conceptualization, Methodology, Investigation, Resources, Data curation, Writing, Review and Editing, Supervision.

## Conflicts of Interest

The authors declare no conflicts of interest.

## Supporting information


**Data S1:** Supporting Information

**Figure S1:** Control sampling site at Montpellier Zoo tropical greenhouse
A. Exterior of the tropical greenhouse. B. View of the giant anteater enclosure. C. General view of the greenhouse interior.

**Figure S2:** Estimated marginal means of within − sample PCR repeatability probability per substrate
Bars represent 95% CI. Asterisks indicate significant differences based on a Tukey test (α = 0.05). Substrates include soil, leaf swabs, and spider webs.

**Table S1:** Marker integration correspondence table
Vertebrate taxa detected by each marker (12S‐V5 and 16Smam) and the consensus taxonomic assignments retained after marker integration. Consensus taxa were selected based on taxonomic resolution, ASV abundance tables, and consistency between markers.

**Table S2:** Sequence analyses summary
Number of reads, samples, and ASVs retained at each step of the bioinformatic filtering and quality control pipeline.

**Table S3:** Detected vertebrate communities per site and substrate
Vertebrate taxa detected at each site and for each substrate after marker integration.

**Table S4:** PCR replicate outcomes per sample and taxon
Number of positive replicates per sample and per taxon after marker integration. For each taxon, the number of positive replicates is provided separately for each marker. Since some taxa were detected by a single marker, the variable *nb_considered_replicates* indicates the total number of replicates considered.

## Data Availability

Genetic data: Raw sequence reads are deposited in Zenodo (https://doi.org/10.5281/zenodo.14843633), Sample metadata: Related metadata can also be found in Zenodo (https://doi.org/10.5281/zenodo.14843633) (including: (a) a metadata file describing the FASTQ files, (b) a metadata file with georeferenced data, (c) raw and filtered abundance tables, and (d) the R script required to reproduce the analyses).
