# Supplementary material for: Spider Webs, Soil or Leaf Swabs to Detect Environmental DNA From Terrestrial Vertebrates: What Is the Best Substrate?
Source: Mol Ecol Resour. 2025 Sep 4;25(8):e70037. doi: 10.1111/1755-0998.70037 (PMC12550491; doi:10.1111/1755-0998.70037)
Supplement: Supplementary file 1 — Data S1: Supporting Information [file MEN-25-e70037-s007.docx]

**Supplementary Information for:**

**Spiderwebs, soil or leaf swabs to detect environmental DNA from terrestrial vertebrates: what is the best substrate?**

**Authors**

Author(s): Berard, Aloïs^1^; Pradel, Julien^1^; Charbonnel, Nathalie^1^ ; Galan, Maxime^1*^

^1^ CBGP, INRAE, CIRAD, IRD, Institut Agro, Univ Montpellier, Montpellier, France

* Corresponding authors : [maxime.galan@inrae.fr](mailto:maxime.galan@inrae.fr)

**Table of Contents** :

[Supplementary Methods S1.1 : Library construction using 2-step PCR (Galan et al. 2018) 1](#_Toc190355531)

[a) Target specific amplification (PCR1) of the 12Sv5 minibarcode: 1](#_Toc190355532)

[b) Target specific amplification (PCR1) of the 16Smam minibarcode: 2](#_Toc190355533)

[c) Unique Dual Indexing (PCR2): 2](#_Toc190355534)

[d) Pooling, size selection & sequencing: 3](#_Toc190355535)

[Supplementary Methods S1.2 : DNA sequence processing & taxonomic assignment 3](#_Toc190355536)

[References: 4](#_Toc190355537)

[Appendix: List of the i5 and i7 sequences (Martin et al 2019): 4](#_Toc190355538)

# Supplementary Methods S1.1 : Library construction using 2-step PCR (Galan et al. 2018)

We performed three technical replicates on each DNA extract in order to control PCR stochasticity for rare DNA targets and to maximize taxa detection. Negative controls for extraction (NCext, 1 per DNA extraction session), PCR (1 NCpcr) and indexing (1 NCindex) were included in the sequencing.

## Target specific amplification (PCR1) of the 12Sv5 minibarcode:

A first amplification step of the short ~100 bp 12Sv5 fragment was carried out using universal primers (12Sv5-forward 5’-TAGAACAGGCTCCTCTAG-3’ and 12Sv5-reverse 5’-TTAGATACCCCACTATGC-3’) described in Riaz et al. 2011 and modified by 5' end overhang Illumina and heterogeneity spacers. The PCR reactions were carried out in a final volume of 10 µL containing 5 µL of Multiplex Master Mix (Qiagen, Hilden, Germany), 0.5 μM of each primer and 2 µL of DNA. The PCR conditions for this first step consisted of initial denaturation at 95°C for 10 min followed by 42 cycles of denaturation at 95°C for 30 s, hybridization at 51°C for 30 s and extension at 72°C for 1 min, and a final extension step at 72°C for 10 min.

12Sv5 forward primer sequences (equimolar mix):

12S-V5-F-V0 TCGTCGGCAGCGTCAGATGTGTATAAGAGACAGTAGAACAGGCTCCTCTAG

12S-V5-F-V1 TCGTCGGCAGCGTCAGATGTGTATAAGAGACAGATAGAACAGGCTCCTCTAG

12S-V5-F-V2 TCGTCGGCAGCGTCAGATGTGTATAAGAGACAGCNTAGAACAGGCTCCTCTAG

12S-V5-F-V3 TCGTCGGCAGCGTCAGATGTGTATAAGAGACAGGCNTAGAACAGGCTCCTCTAG

12S-V5-F-V4 TCGTCGGCAGCGTCAGATGTGTATAAGAGACAGNGCCTAGAACAGGCTCCTCTAG

12Sv5 reverse primer sequence (equimolar mix):

12S-V5-R-V0 GTCTCGTGGGCTCGGAGATGTGTATAAGAGACAGTTAGATACCCCACTATGC

12S-V5-R-V1 GTCTCGTGGGCTCGGAGATGTGTATAAGAGACAGATTAGATACCCCACTATGC

12S-V5-R-V2 GTCTCGTGGGCTCGGAGATGTGTATAAGAGACAGCATTAGATACCCCACTATGC

12S-V5-R-V3 GTCTCGTGGGCTCGGAGATGTGTATAAGAGACAGGCGTTAGATACCCCACTATGC

12S-V5-R-V4 GTCTCGTGGGCTCGGAGATGTGTATAAGAGACAGNGCCTTAGATACCCCACTATGC

## Target specific amplification (PCR1) of the 16Smam minibarcode:

A first amplification step of the short~94 bp 16S rRNA mitochondrial fragment was carried out using mammalian primers (16Smam1 5’- CGGTTGGGGTGACCTCGGA -3’ and 16Smam2 5’- GCTGTTATCCCTAGGGTAACT -3’) described in Taylor et al. 1996 and modified by 5' end overhang Illumina and heterogeneity spacers. The PCR reactions were carried out in a final volume of 10 µL containing 5 µL of Multiplex Master Mix (Qiagen, Hilden, Germany), 0.5 μM of each primer and 2 µL of DNA. The PCR conditions for this first step consisted of initial denaturation at 95°C for 10 min followed by 42 cycles of denaturation at 95°C for 30 s, hybridization at 55°C for 30 s and extension at 72°C for 45 s, and a final extension step at 72°C for 10 min.

16Smam1 forward primer sequences (equimolar mix):

16Smam1-F-V0 TCGTCGGCAGCGTCAGATGTGTATAAGAGACAGCGGTTGGGGTGACCTCGGA

16Smam1-F-V1 TCGTCGGCAGCGTCAGATGTGTATAAGAGACAGACGGTTGGGGTGACCTCGGA

16Smam1-F-V2 TCGTCGGCAGCGTCAGATGTGTATAAGAGACAGTNCGGTTGGGGTGACCTCGGA

16Smam1-F-V3 TCGTCGGCAGCGTCAGATGTGTATAAGAGACAGGATCGGTTGGGGTGACCTCGGA

16Smam1-F-V4 TCGTCGGCAGCGTCAGATGTGTATAAGAGACAGNTATCGGTTGGGGTGACCTCGGA

16Smam2 reverse primer sequence (equimolar mix):

16Smam2-R-V0 GTCTCGTGGGCTCGGAGATGTGTATAAGAGACAGGCTGTTATCCCTAGGGTAACT

16Smam2-R-V1 GTCTCGTGGGCTCGGAGATGTGTATAAGAGACAGTGCTGTTATCCCTAGGGTAACT

16Smam2-R-V2 GTCTCGTGGGCTCGGAGATGTGTATAAGAGACAGANGCTGTTATCCCTAGGGTAACT

16Smam2-R-V3 GTCTCGTGGGCTCGGAGATGTGTATAAGAGACAGCANGCTGTTATCCCTAGGGTAACT

16Smam2-R-V4 GTCTCGTGGGCTCGGAGATGTGTATAAGAGACAGNTAAGCTGTTATCCCTAGGGTAACT

## Unique Dual Indexing (PCR2):

A second PCR step was performed to add individual-specific multiplexing tags (called index i5 and index i7), consisting of short 9 bp sample-specific sequences (Martin et al 2019) and the Illumina adapters (called P5 and P7) at the 5' ends of each amplified DNA fragment to the first PCR1. As all the PCR2 products were mixed together (multiplexing) for MiSeq sequencing, dual-indexes made it possible to identify the origin of the sequences and reassign them to each sample (demultiplexing). Each i5 and i7 indexes were used for a unique PCR replicate (i.e. unique dual indexes) to reduce the index-hopping and make sure that libraries were sequence and demultiplex with the highest accuracy (Kircher et al 2012). This second PCR was carried out in a total volume of 10 µL containing 5 µL of Multiplex kit (Qiagen, Germany), 0.7 μM of each primer, and 2 µL of products from the first PCR for each sample. PCR conditions consisted of an initial denaturation step at 95°C for 15 min followed by 8 cycles of denaturation at 95°C for 40 s, hybridization at 55°C for 45 s and extension at 72°C for 2 min, and a final extension step at 72°C for 10 min.

Forward indexed i5 primer PCR2 sequence (i5 & i7 sequences are listed in Appendix):

AATGATACGGCGACCACCGAGATCTACAC<i5>TCGTCGGCAGCGTC

Reverse indexed i7 primer PCR2 sequence:

CAAGCAGAAGACGGCATACGAGAT<i7>GTCTCGTGGGCTCGG

## Pooling, size selection & sequencing:

The volume-to-volume mixes (one for each minibarcode) of all the PCR products (4 µL per sample) were screened based on fragment length resulting from excision on 1.25% agarose gel. The pools of specific PCR products were cut to the expected sizes (~277 bp for 12Sv5 and ~276 bp for 16Smam, corresponding to the size of the amplicon, including the gene-specific primers, sequencing primers, indexes and adaptors) under UV light. The resulting libraries were purified with the PCR purification kit (Macherey-Nagel, Germany), quantified by quantitative PCR (KAPA kit, Kapa Biosciences), then sequenced on a MiSeq sequencer with a 300v2 kit (Illumina, USA).

# Supplementary Methods S1.2 : DNA sequence processing & taxonomic assignment

We processed the paired-end sequencing with the FROGS pipeline (Escudié et al 2017) available at [https://github.com/geral dinepascal/ FROGS.git](https://github.com/geral%20dinepascal/%20FROGS.git). We preliminarily used a Shell home-made script (<https://zenodo.org/records/10728322>) to merge pair sequences into contigs with FLASH v. 1.2.11 (Magoc et Salzberg 2011), and trim primers with cutadapt v. 2.10 (Martin 2011). With FROGS pipeline, we then filtered sequences by length (expected value of 100bp +/- 22bp for 12Sv5 and 110bp +/- 50bp for 16Smam), dereplicated sequences, removed chimeras using the algorithm of Edgar et al. (2011) implemented in VSEARCH v. 2.17.0 and clustered sequences with SWARM v. 3.0.0 using a local clustering threshold with default d-value (maximum number of differences between sequences in each aggregation swarm steps d=1) and the swarm fastidious option to refine the clustering (Mahé et al 2014). When using the fastidious method with d=1, swarm aims to produce clusters centred around real biological sequences, where clusters represent sequence variants.

We discarded the singleton ASVs (Amplicon Sequence Variants) *i.e.,* showing less than 2 reads in the sequencing run and/or not shared by at least 2 amplicon libraries. Since the remaining chimera were visually detected in the abundance table (sequences formed by two more abundant sequences of the same sample), we used the *isBimeraDeNovo* function from the dada2 package (Callahan et al., 2019) to detect and remove the residual chimeras. Filtering for false positives was carried out as proposed by Galan et al. (2016): we discarded positive results associated with sequence counts below an ASV-specific threshold, which checked for cross-contamination between samples using the negative controls.

Taxonomic affiliations for each ASV were returned using BLASTN (Altschul et al 1990) and the NCBI Core Nucleotide database (core_nt) on GenBank (Benson et al 2008). The identification was considered as ‘valid’ from a similarity threshold of 98 to 100%. Below that threshold, the ASVs were considered unidentified. When multiple assignments shared equivalent best matches, ASV was assigned to the common taxonomic rank unless only one of the matching species was known to occur within the study area, in which case that species was retained. Finally, ASVs affiliated to the same taxa were grouped together and unassigned ASVs were discarded. ASVs with the same taxonomic affiliation were merged. Finally, for each marker, the technical replicates were combined by summing reads per sample.

# References:

Altschul, S. F., Gish, W., Miller, W., Myers, E. W., & Lipman, D. J. (1990). Basic local alignment search tool. Journal of Molecular Biology, 215, 403–410. [https://doi.org/10.1016/S0022 -2836(05)80360 -2](https://doi.org/10.1016/S0022%20-2836(05)80360%20-2)

Benson, D. A., Karsch-Mizrachi, I., Lipman, D. J., Ostell, J., & Wheeler, D.L. (2008). GenBank. Nucleic Acids Research, 36, D25–D30. <https://doi.org/10.1093/nar/gkm929>

Callahan, B. J., Wong, J., Heiner, C., Oh, S., Theriot, C. M., Gulati, A. S., McGill, S. K., & Dougherty, M. K. (2019). High-throughput amplicon sequencing of the full-length 16S rRNA gene with single-nucleotide resolution. Nucleic Acids Research, 47(18), e103. <https://doi.org/10.1093/nar/gkz569>

Edgar, R. C., Haas, B. J., Clemente, J. C., Quince, C., & Knight, R. (2011). UCHIME improves sensitivity and speed of chimera detection. Bioinformatics, 27, 2194–2200. [https://doi.org/10.1093/bioin formatics/btr381](https://doi.org/10.1093/bioin%20formatics/btr381)

Escudié, F., Auer, L., Bernard, M., Mariadassou, M., Cauquil, L., Vidal, K., Maman, S., Hernandez-Raquet, G., Combes, S., & Pascal, G. (2018). FROGS: Find, rapidly, OTUs with galaxy solution. Bioinformatics, 34(8), 1287–1294. [https://doi.org/10.1093/bioin forma tics/btx791](https://doi.org/10.1093/bioin%20forma%20tics/btx791)

Galan, M., Razzauti, M., Bard, E., Bernard, M., Brouat, C., Charbonnel, N., … Cosson, J.-F. (2016). 16S rRNA amplicon sequencing for epidemiological surveys of bacteria in wildlife. mSystems, 1, e00032-16. [https://doi.org/10.1128/mSyst ems.00032 -16](https://doi.org/10.1128/mSyst%20ems.00032%20-16)

Galan, M., Pons, J.-B., Tournayre, O., Pierre, É., Leuchtmann, M., Pontier, D., & Charbonnel, N. (2018). Metabarcoding for the parallel identification of several hundred predators and their prey: Application to bat species diet analysis. Molecular Ecology Resources, 18, 474–489. <https://doi.org/10.1111/1755-0998.12749>

Magoc, T., & Salzberg, S. L. (2011). FLASH: Fast length adjustment of short reads to improve genome assemblies. Bioinformatics, 27(21), 2957–2963. [https://doi.org/10.1093/bioin forma tics/btr507](https://doi.org/10.1093/bioin%20forma%20tics/btr507)

Mahé, F., Rognes, T., Quince, C., de Vargas, C., & Dunthorn, M. (2014). Swarm: Robust and fast clustering method for amplicon-based studies. PeerJ, 2, e593. <https://doi.org/10.7717/peerj.593>

Martin, M. (2011). Cutadapt removes adapter sequences from high-throughput sequencing reads. EMBnet.Journal, 17(1), 10. [https://doi.org/10.14806/ ej.17.1.200](https://doi.org/10.14806/%20ej.17.1.200)

Martin, J.-F. (2019). Creating error-proof indexes for high throughput sequencing. Zenodo, <https://doi.org/10.5281/zenodo.3350207>

Riaz, T., Shehzad, W., Viari, A., Pompanon, F., Taberlet, P., & Coissac, E. (2011). ecoPrimers: inference of new DNA barcode markers from whole genome sequence analysis. Nucleic Acids Research, 39(21), e145

Taylor, P. G. (1996). Reproducibility of ancient DNA sequences from extinct Pleistocene fauna. Molecular Biology and Evolution, 13(1), 283–285.

# Appendix: List of the i5 and i7 sequences (Martin et al 2019):

| Plate | Well | Index name | Index sequence to fill the sample sheet | Indexed primer PCR2 sequences |
| --- | --- | --- | --- | --- |
| 1 | A1 | I5A001 | GCTCACTTG | AATGATACGGCGACCACCGAGATCTACACGCTCACTTGTCGTCGGCAGCGTC |
| 1 | B1 | I5A002 | ACGGAGTTC | AATGATACGGCGACCACCGAGATCTACACACGGAGTTCTCGTCGGCAGCGTC |
| 1 | C1 | I5A003 | CTTAGTCTG | AATGATACGGCGACCACCGAGATCTACACCTTAGTCTGTCGTCGGCAGCGTC |
| 1 | D1 | I5A004 | ATTCGGTTG | AATGATACGGCGACCACCGAGATCTACACATTCGGTTGTCGTCGGCAGCGTC |
| 1 | E1 | I5A005 | GAGGTATCA | AATGATACGGCGACCACCGAGATCTACACGAGGTATCATCGTCGGCAGCGTC |
| 1 | F1 | I5A006 | ACCGGTATA | AATGATACGGCGACCACCGAGATCTACACACCGGTATATCGTCGGCAGCGTC |
| 1 | G1 | I5A007 | GAGATAGTG | AATGATACGGCGACCACCGAGATCTACACGAGATAGTGTCGTCGGCAGCGTC |
| 1 | H1 | I5A008 | GGTAGATCA | AATGATACGGCGACCACCGAGATCTACACGGTAGATCATCGTCGGCAGCGTC |
| 1 | A2 | I5A009 | TTGGAGAGA | AATGATACGGCGACCACCGAGATCTACACTTGGAGAGATCGTCGGCAGCGTC |
| 1 | B2 | I5A010 | AACCTCGAA | AATGATACGGCGACCACCGAGATCTACACAACCTCGAATCGTCGGCAGCGTC |
| 1 | C2 | I5A011 | TGGACGGAA | AATGATACGGCGACCACCGAGATCTACACTGGACGGAATCGTCGGCAGCGTC |
| 1 | D2 | I5A012 | GATGTCTCG | AATGATACGGCGACCACCGAGATCTACACGATGTCTCGTCGTCGGCAGCGTC |
| 1 | E2 | I5A013 | GAATACTCG | AATGATACGGCGACCACCGAGATCTACACGAATACTCGTCGTCGGCAGCGTC |
| 1 | F2 | I5A014 | GTGTGCTGA | AATGATACGGCGACCACCGAGATCTACACGTGTGCTGATCGTCGGCAGCGTC |
| 1 | G2 | I5A015 | GGTTCTCCA | AATGATACGGCGACCACCGAGATCTACACGGTTCTCCATCGTCGGCAGCGTC |
| 1 | H2 | I5A016 | ATCGTCTGT | AATGATACGGCGACCACCGAGATCTACACATCGTCTGTTCGTCGGCAGCGTC |
| 1 | A3 | I5A017 | GAACAAGAG | AATGATACGGCGACCACCGAGATCTACACGAACAAGAGTCGTCGGCAGCGTC |
| 1 | B3 | I5A018 | GTTCTTCGA | AATGATACGGCGACCACCGAGATCTACACGTTCTTCGATCGTCGGCAGCGTC |
| 1 | C3 | I5A019 | ATACCAACC | AATGATACGGCGACCACCGAGATCTACACATACCAACCTCGTCGGCAGCGTC |
| 1 | D3 | I5A020 | CGTGTTCCA | AATGATACGGCGACCACCGAGATCTACACCGTGTTCCATCGTCGGCAGCGTC |
| 1 | E3 | I5A021 | CCTTCCTAA | AATGATACGGCGACCACCGAGATCTACACCCTTCCTAATCGTCGGCAGCGTC |
| 1 | F3 | I5A022 | CTTCACACA | AATGATACGGCGACCACCGAGATCTACACCTTCACACATCGTCGGCAGCGTC |
| 1 | G3 | I5A023 | GGATATCTG | AATGATACGGCGACCACCGAGATCTACACGGATATCTGTCGTCGGCAGCGTC |
| 1 | H3 | I5A024 | TGGCCTATA | AATGATACGGCGACCACCGAGATCTACACTGGCCTATATCGTCGGCAGCGTC |
| 1 | A4 | I5A025 | ATTGTCAGG | AATGATACGGCGACCACCGAGATCTACACATTGTCAGGTCGTCGGCAGCGTC |
| 1 | B4 | I5A026 | GTGAAGGAA | AATGATACGGCGACCACCGAGATCTACACGTGAAGGAATCGTCGGCAGCGTC |
| 1 | C4 | I5A027 | CCGACCATT | AATGATACGGCGACCACCGAGATCTACACCCGACCATTTCGTCGGCAGCGTC |
| 1 | D4 | I5A028 | CCGTCTATA | AATGATACGGCGACCACCGAGATCTACACCCGTCTATATCGTCGGCAGCGTC |
| 1 | E4 | I5A029 | CTCTCTACC | AATGATACGGCGACCACCGAGATCTACACCTCTCTACCTCGTCGGCAGCGTC |
| 1 | F4 | I5A030 | CCTGTCTTC | AATGATACGGCGACCACCGAGATCTACACCCTGTCTTCTCGTCGGCAGCGTC |
| 1 | G4 | I5A031 | TGACTTGTG | AATGATACGGCGACCACCGAGATCTACACTGACTTGTGTCGTCGGCAGCGTC |
| 1 | H4 | I5A032 | TAGCAACAC | AATGATACGGCGACCACCGAGATCTACACTAGCAACACTCGTCGGCAGCGTC |
| 1 | A5 | I5A033 | TGCAGACAA | AATGATACGGCGACCACCGAGATCTACACTGCAGACAATCGTCGGCAGCGTC |
| 1 | B5 | I5A034 | GACTCACAA | AATGATACGGCGACCACCGAGATCTACACGACTCACAATCGTCGGCAGCGTC |
| 1 | C5 | I5A035 | AGACCAGAA | AATGATACGGCGACCACCGAGATCTACACAGACCAGAATCGTCGGCAGCGTC |
| 1 | D5 | I5A036 | TACTGTTGC | AATGATACGGCGACCACCGAGATCTACACTACTGTTGCTCGTCGGCAGCGTC |
| 1 | E5 | I5A037 | TTGTGATGG | AATGATACGGCGACCACCGAGATCTACACTTGTGATGGTCGTCGGCAGCGTC |
| 1 | F5 | I5A038 | GGTTCAGAA | AATGATACGGCGACCACCGAGATCTACACGGTTCAGAATCGTCGGCAGCGTC |
| 1 | G5 | I5A039 | TTGGTGTCA | AATGATACGGCGACCACCGAGATCTACACTTGGTGTCATCGTCGGCAGCGTC |
| 1 | H5 | I5A040 | GTACACACG | AATGATACGGCGACCACCGAGATCTACACGTACACACGTCGTCGGCAGCGTC |
| 1 | A6 | I5A041 | ACTTGCTTC | AATGATACGGCGACCACCGAGATCTACACACTTGCTTCTCGTCGGCAGCGTC |
| 1 | B6 | I5A042 | ACCTCAGCA | AATGATACGGCGACCACCGAGATCTACACACCTCAGCATCGTCGGCAGCGTC |
| 1 | C6 | I5A043 | CTAACTCCA | AATGATACGGCGACCACCGAGATCTACACCTAACTCCATCGTCGGCAGCGTC |
| 1 | D6 | I5A044 | AATTCCGGA | AATGATACGGCGACCACCGAGATCTACACAATTCCGGATCGTCGGCAGCGTC |
| 1 | E6 | I5A045 | GAAGCCTCT | AATGATACGGCGACCACCGAGATCTACACGAAGCCTCTTCGTCGGCAGCGTC |
| 1 | F6 | I5A046 | TTGCGGTCT | AATGATACGGCGACCACCGAGATCTACACTTGCGGTCTTCGTCGGCAGCGTC |
| 1 | G6 | I5A047 | AAGTAGACG | AATGATACGGCGACCACCGAGATCTACACAAGTAGACGTCGTCGGCAGCGTC |
| 1 | H6 | I5A048 | GGTTGACTT | AATGATACGGCGACCACCGAGATCTACACGGTTGACTTTCGTCGGCAGCGTC |
| 1 | A7 | I5A049 | AACCACACA | AATGATACGGCGACCACCGAGATCTACACAACCACACATCGTCGGCAGCGTC |
| 1 | B7 | I5A050 | GCTTCTCTG | AATGATACGGCGACCACCGAGATCTACACGCTTCTCTGTCGTCGGCAGCGTC |
| 1 | C7 | I5A051 | GCGCTCTAA | AATGATACGGCGACCACCGAGATCTACACGCGCTCTAATCGTCGGCAGCGTC |
| 1 | D7 | I5A052 | GGAATACGG | AATGATACGGCGACCACCGAGATCTACACGGAATACGGTCGTCGGCAGCGTC |
| 1 | E7 | I5A053 | CGAGGATTA | AATGATACGGCGACCACCGAGATCTACACCGAGGATTATCGTCGGCAGCGTC |
| 1 | F7 | I5A054 | TCTCATCTG | AATGATACGGCGACCACCGAGATCTACACTCTCATCTGTCGTCGGCAGCGTC |
| 1 | G7 | I5A055 | ACGCAACAA | AATGATACGGCGACCACCGAGATCTACACACGCAACAATCGTCGGCAGCGTC |
| 1 | H7 | I5A056 | TTAACGTGG | AATGATACGGCGACCACCGAGATCTACACTTAACGTGGTCGTCGGCAGCGTC |
| 1 | A8 | I5A057 | CAATCGGAA | AATGATACGGCGACCACCGAGATCTACACCAATCGGAATCGTCGGCAGCGTC |
| 1 | B8 | I5A058 | CAATGTGTG | AATGATACGGCGACCACCGAGATCTACACCAATGTGTGTCGTCGGCAGCGTC |
| 1 | C8 | I5A059 | CACGCATAA | AATGATACGGCGACCACCGAGATCTACACCACGCATAATCGTCGGCAGCGTC |
| 1 | D8 | I5A060 | CGTGGTATA | AATGATACGGCGACCACCGAGATCTACACCGTGGTATATCGTCGGCAGCGTC |
| 1 | E8 | I5A061 | ACAATGCCG | AATGATACGGCGACCACCGAGATCTACACACAATGCCGTCGTCGGCAGCGTC |
| 1 | F8 | I5A062 | TCCACGCAA | AATGATACGGCGACCACCGAGATCTACACTCCACGCAATCGTCGGCAGCGTC |
| 1 | G8 | I5A063 | CTGTGAGAA | AATGATACGGCGACCACCGAGATCTACACCTGTGAGAATCGTCGGCAGCGTC |
| 1 | H8 | I5A064 | TGGTTGGTA | AATGATACGGCGACCACCGAGATCTACACTGGTTGGTATCGTCGGCAGCGTC |
| 1 | A9 | I5A065 | TGAACCTCA | AATGATACGGCGACCACCGAGATCTACACTGAACCTCATCGTCGGCAGCGTC |
| 1 | B9 | I5A066 | CACAGACAC | AATGATACGGCGACCACCGAGATCTACACCACAGACACTCGTCGGCAGCGTC |
| 1 | C9 | I5A067 | AGCCTGTGT | AATGATACGGCGACCACCGAGATCTACACAGCCTGTGTTCGTCGGCAGCGTC |
| 1 | D9 | I5A068 | GTATTGGAG | AATGATACGGCGACCACCGAGATCTACACGTATTGGAGTCGTCGGCAGCGTC |
| 1 | E9 | I5A069 | TAACGAGCC | AATGATACGGCGACCACCGAGATCTACACTAACGAGCCTCGTCGGCAGCGTC |
| 1 | F9 | I5A070 | GGCGTCTTA | AATGATACGGCGACCACCGAGATCTACACGGCGTCTTATCGTCGGCAGCGTC |
| 1 | G9 | I5A071 | ATTGTGCTC | AATGATACGGCGACCACCGAGATCTACACATTGTGCTCTCGTCGGCAGCGTC |
| 1 | H9 | I5A072 | AAGAGCTTC | AATGATACGGCGACCACCGAGATCTACACAAGAGCTTCTCGTCGGCAGCGTC |
| 1 | A10 | I5A073 | AAGCTTAGG | AATGATACGGCGACCACCGAGATCTACACAAGCTTAGGTCGTCGGCAGCGTC |
| 1 | B10 | I5A074 | GACGACCTA | AATGATACGGCGACCACCGAGATCTACACGACGACCTATCGTCGGCAGCGTC |
| 1 | C10 | I5A075 | GCCGATTAA | AATGATACGGCGACCACCGAGATCTACACGCCGATTAATCGTCGGCAGCGTC |
| 1 | D10 | I5A076 | TGCGTGGAA | AATGATACGGCGACCACCGAGATCTACACTGCGTGGAATCGTCGGCAGCGTC |
| 1 | E10 | I5A077 | GTTCACCTA | AATGATACGGCGACCACCGAGATCTACACGTTCACCTATCGTCGGCAGCGTC |
| 1 | F10 | I5A078 | TGACCACTT | AATGATACGGCGACCACCGAGATCTACACTGACCACTTTCGTCGGCAGCGTC |
| 1 | G10 | I5A079 | GCGGCATAA | AATGATACGGCGACCACCGAGATCTACACGCGGCATAATCGTCGGCAGCGTC |
| 1 | H10 | I5A080 | CACGTGGTT | AATGATACGGCGACCACCGAGATCTACACCACGTGGTTTCGTCGGCAGCGTC |
| 1 | A11 | I5A081 | ACAGGACAA | AATGATACGGCGACCACCGAGATCTACACACAGGACAATCGTCGGCAGCGTC |
| 1 | B11 | I5A082 | AGGTATTCG | AATGATACGGCGACCACCGAGATCTACACAGGTATTCGTCGTCGGCAGCGTC |
| 1 | C11 | I5A083 | TAGGTTCCA | AATGATACGGCGACCACCGAGATCTACACTAGGTTCCATCGTCGGCAGCGTC |
| 1 | D11 | I5A084 | TGGTAAGTG | AATGATACGGCGACCACCGAGATCTACACTGGTAAGTGTCGTCGGCAGCGTC |
| 1 | E11 | I5A085 | GCGTTGTGT | AATGATACGGCGACCACCGAGATCTACACGCGTTGTGTTCGTCGGCAGCGTC |
| 1 | F11 | I5A086 | GCAACAGAA | AATGATACGGCGACCACCGAGATCTACACGCAACAGAATCGTCGGCAGCGTC |
| 1 | G11 | I5A087 | TCCTGAGAA | AATGATACGGCGACCACCGAGATCTACACTCCTGAGAATCGTCGGCAGCGTC |
| 1 | H11 | I5A088 | CTTATACGG | AATGATACGGCGACCACCGAGATCTACACCTTATACGGTCGTCGGCAGCGTC |
| 1 | A12 | I5A089 | TTACGCTCC | AATGATACGGCGACCACCGAGATCTACACTTACGCTCCTCGTCGGCAGCGTC |
| 1 | B12 | I5A090 | GCCACCATA | AATGATACGGCGACCACCGAGATCTACACGCCACCATATCGTCGGCAGCGTC |
| 1 | C12 | I5A091 | GGTCAACAA | AATGATACGGCGACCACCGAGATCTACACGGTCAACAATCGTCGGCAGCGTC |
| 1 | D12 | I5A092 | CCGGTTCAA | AATGATACGGCGACCACCGAGATCTACACCCGGTTCAATCGTCGGCAGCGTC |
| 1 | E12 | I5A093 | ACCACAGTG | AATGATACGGCGACCACCGAGATCTACACACCACAGTGTCGTCGGCAGCGTC |
| 1 | F12 | I5A094 | AAGCGAGAA | AATGATACGGCGACCACCGAGATCTACACAAGCGAGAATCGTCGGCAGCGTC |
| 1 | G12 | I5A095 | AAGACGCCA | AATGATACGGCGACCACCGAGATCTACACAAGACGCCATCGTCGGCAGCGTC |
| 1 | H12 | I5A096 | AGGTTGTGA | AATGATACGGCGACCACCGAGATCTACACAGGTTGTGATCGTCGGCAGCGTC |
| 2 | A1 | I5B097 | GATCCTCCT | AATGATACGGCGACCACCGAGATCTACACGATCCTCCTTCGTCGGCAGCGTC |
| 2 | B1 | I5B098 | TTCGTTAGG | AATGATACGGCGACCACCGAGATCTACACTTCGTTAGGTCGTCGGCAGCGTC |
| 2 | C1 | I5B099 | CTCAACCAA | AATGATACGGCGACCACCGAGATCTACACCTCAACCAATCGTCGGCAGCGTC |
| 2 | D1 | I5B100 | GCTCCTATC | AATGATACGGCGACCACCGAGATCTACACGCTCCTATCTCGTCGGCAGCGTC |
| 2 | E1 | I5B101 | CTCGTTCTC | AATGATACGGCGACCACCGAGATCTACACCTCGTTCTCTCGTCGGCAGCGTC |
| 2 | F1 | I5B102 | CTTCTGAGG | AATGATACGGCGACCACCGAGATCTACACCTTCTGAGGTCGTCGGCAGCGTC |
| 2 | G1 | I5B103 | ACTAAGCCA | AATGATACGGCGACCACCGAGATCTACACACTAAGCCATCGTCGGCAGCGTC |
| 2 | H1 | I5B104 | TCCATTACG | AATGATACGGCGACCACCGAGATCTACACTCCATTACGTCGTCGGCAGCGTC |
| 2 | A2 | I5B105 | CTTGTTAGC | AATGATACGGCGACCACCGAGATCTACACCTTGTTAGCTCGTCGGCAGCGTC |
| 2 | B2 | I5B106 | ATTCCACGG | AATGATACGGCGACCACCGAGATCTACACATTCCACGGTCGTCGGCAGCGTC |
| 2 | C2 | I5B107 | TGGCGATTG | AATGATACGGCGACCACCGAGATCTACACTGGCGATTGTCGTCGGCAGCGTC |
| 2 | D2 | I5B108 | AGGAGCGAA | AATGATACGGCGACCACCGAGATCTACACAGGAGCGAATCGTCGGCAGCGTC |
| 2 | E2 | I5B109 | GTAAGAGCA | AATGATACGGCGACCACCGAGATCTACACGTAAGAGCATCGTCGGCAGCGTC |
| 2 | F2 | I5B110 | CTAAGGCCT | AATGATACGGCGACCACCGAGATCTACACCTAAGGCCTTCGTCGGCAGCGTC |
| 2 | G2 | I5B111 | AATCATCGG | AATGATACGGCGACCACCGAGATCTACACAATCATCGGTCGTCGGCAGCGTC |
| 2 | H2 | I5B112 | ATTCTGGCC | AATGATACGGCGACCACCGAGATCTACACATTCTGGCCTCGTCGGCAGCGTC |
| 2 | A3 | I5B113 | CGGACACAA | AATGATACGGCGACCACCGAGATCTACACCGGACACAATCGTCGGCAGCGTC |
| 2 | B3 | I5B114 | GAACCGTAA | AATGATACGGCGACCACCGAGATCTACACGAACCGTAATCGTCGGCAGCGTC |
| 2 | C3 | I5B115 | TGTGCCACA | AATGATACGGCGACCACCGAGATCTACACTGTGCCACATCGTCGGCAGCGTC |
| 2 | D3 | I5B116 | GAGTAACCA | AATGATACGGCGACCACCGAGATCTACACGAGTAACCATCGTCGGCAGCGTC |
| 2 | E3 | I5B117 | TTAGCTTGC | AATGATACGGCGACCACCGAGATCTACACTTAGCTTGCTCGTCGGCAGCGTC |
| 2 | F3 | I5B118 | GACACGGAA | AATGATACGGCGACCACCGAGATCTACACGACACGGAATCGTCGGCAGCGTC |
| 2 | G3 | I5B119 | CTCCACTGA | AATGATACGGCGACCACCGAGATCTACACCTCCACTGATCGTCGGCAGCGTC |
| 2 | H3 | I5B120 | CTTGGTGAA | AATGATACGGCGACCACCGAGATCTACACCTTGGTGAATCGTCGGCAGCGTC |
| 2 | A4 | I5B121 | GCAACATCT | AATGATACGGCGACCACCGAGATCTACACGCAACATCTTCGTCGGCAGCGTC |
| 2 | B4 | I5B122 | TCTACACAG | AATGATACGGCGACCACCGAGATCTACACTCTACACAGTCGTCGGCAGCGTC |
| 2 | C4 | I5B123 | AAGTCTGCG | AATGATACGGCGACCACCGAGATCTACACAAGTCTGCGTCGTCGGCAGCGTC |
| 2 | D4 | I5B124 | AGCACATCA | AATGATACGGCGACCACCGAGATCTACACAGCACATCATCGTCGGCAGCGTC |
| 2 | E4 | I5B125 | CACAAGTCC | AATGATACGGCGACCACCGAGATCTACACCACAAGTCCTCGTCGGCAGCGTC |
| 2 | F4 | I5B126 | GTCCAAGAA | AATGATACGGCGACCACCGAGATCTACACGTCCAAGAATCGTCGGCAGCGTC |
| 2 | G4 | I5B127 | TGTTGTCTG | AATGATACGGCGACCACCGAGATCTACACTGTTGTCTGTCGTCGGCAGCGTC |
| 2 | H4 | I5B128 | GTGGTTCCT | AATGATACGGCGACCACCGAGATCTACACGTGGTTCCTTCGTCGGCAGCGTC |
| 2 | A5 | I5B129 | CGCGATGAA | AATGATACGGCGACCACCGAGATCTACACCGCGATGAATCGTCGGCAGCGTC |
| 2 | B5 | I5B130 | TGAGGAGGT | AATGATACGGCGACCACCGAGATCTACACTGAGGAGGTTCGTCGGCAGCGTC |
| 2 | C5 | I5B131 | TAAGCCGCA | AATGATACGGCGACCACCGAGATCTACACTAAGCCGCATCGTCGGCAGCGTC |
| 2 | D5 | I5B132 | AGCGGATTG | AATGATACGGCGACCACCGAGATCTACACAGCGGATTGTCGTCGGCAGCGTC |
| 2 | E5 | I5B133 | CCGCTTGTT | AATGATACGGCGACCACCGAGATCTACACCCGCTTGTTTCGTCGGCAGCGTC |
| 2 | F5 | I5B134 | TTGAGATCC | AATGATACGGCGACCACCGAGATCTACACTTGAGATCCTCGTCGGCAGCGTC |
| 2 | G5 | I5B135 | TTGAAGCTG | AATGATACGGCGACCACCGAGATCTACACTTGAAGCTGTCGTCGGCAGCGTC |
| 2 | H5 | I5B136 | GAATTAGCC | AATGATACGGCGACCACCGAGATCTACACGAATTAGCCTCGTCGGCAGCGTC |
| 2 | A6 | I5B137 | TGAGTCTCC | AATGATACGGCGACCACCGAGATCTACACTGAGTCTCCTCGTCGGCAGCGTC |
| 2 | B6 | I5B138 | CTTATGCCA | AATGATACGGCGACCACCGAGATCTACACCTTATGCCATCGTCGGCAGCGTC |
| 2 | C6 | I5B139 | GAATGGCGT | AATGATACGGCGACCACCGAGATCTACACGAATGGCGTTCGTCGGCAGCGTC |
| 2 | D6 | I5B140 | GTTCCAGTT | AATGATACGGCGACCACCGAGATCTACACGTTCCAGTTTCGTCGGCAGCGTC |
| 2 | E6 | I5B141 | CAACCTTGG | AATGATACGGCGACCACCGAGATCTACACCAACCTTGGTCGTCGGCAGCGTC |
| 2 | F6 | I5B142 | CCTTAACTG | AATGATACGGCGACCACCGAGATCTACACCCTTAACTGTCGTCGGCAGCGTC |
| 2 | G6 | I5B143 | ATTAAGGCG | AATGATACGGCGACCACCGAGATCTACACATTAAGGCGTCGTCGGCAGCGTC |
| 2 | H6 | I5B144 | TGAGGTGAA | AATGATACGGCGACCACCGAGATCTACACTGAGGTGAATCGTCGGCAGCGTC |
| 2 | A7 | I5B145 | CACACATGG | AATGATACGGCGACCACCGAGATCTACACCACACATGGTCGTCGGCAGCGTC |
| 2 | B7 | I5B146 | ATGATGCGG | AATGATACGGCGACCACCGAGATCTACACATGATGCGGTCGTCGGCAGCGTC |
| 2 | C7 | I5B147 | TGGACTTCC | AATGATACGGCGACCACCGAGATCTACACTGGACTTCCTCGTCGGCAGCGTC |
| 2 | D7 | I5B148 | GTAGAGTGA | AATGATACGGCGACCACCGAGATCTACACGTAGAGTGATCGTCGGCAGCGTC |
| 2 | E7 | I5B149 | TCCACTCTC | AATGATACGGCGACCACCGAGATCTACACTCCACTCTCTCGTCGGCAGCGTC |
| 2 | F7 | I5B150 | GTTAGGTGC | AATGATACGGCGACCACCGAGATCTACACGTTAGGTGCTCGTCGGCAGCGTC |
| 2 | G7 | I5B151 | AGAGTGGTA | AATGATACGGCGACCACCGAGATCTACACAGAGTGGTATCGTCGGCAGCGTC |
| 2 | H7 | I5B152 | GAAGGTCAA | AATGATACGGCGACCACCGAGATCTACACGAAGGTCAATCGTCGGCAGCGTC |
| 2 | A8 | I5B153 | TAGGCGTGA | AATGATACGGCGACCACCGAGATCTACACTAGGCGTGATCGTCGGCAGCGTC |
| 2 | B8 | I5B154 | CTCCTTATG | AATGATACGGCGACCACCGAGATCTACACCTCCTTATGTCGTCGGCAGCGTC |
| 2 | C8 | I5B155 | AATTGAGCG | AATGATACGGCGACCACCGAGATCTACACAATTGAGCGTCGTCGGCAGCGTC |
| 2 | D8 | I5B156 | ACGGCTCTT | AATGATACGGCGACCACCGAGATCTACACACGGCTCTTTCGTCGGCAGCGTC |
| 2 | E8 | I5B157 | ACTCTTACG | AATGATACGGCGACCACCGAGATCTACACACTCTTACGTCGTCGGCAGCGTC |
| 2 | F8 | I5B158 | AGGATAAGG | AATGATACGGCGACCACCGAGATCTACACAGGATAAGGTCGTCGGCAGCGTC |
| 2 | G8 | I5B159 | ACTACCGTT | AATGATACGGCGACCACCGAGATCTACACACTACCGTTTCGTCGGCAGCGTC |
| 2 | H8 | I5B160 | AGGCCTCAA | AATGATACGGCGACCACCGAGATCTACACAGGCCTCAATCGTCGGCAGCGTC |
| 2 | A9 | I5B161 | ATTCCTCCA | AATGATACGGCGACCACCGAGATCTACACATTCCTCCATCGTCGGCAGCGTC |
| 2 | B9 | I5B162 | TCTATGGAG | AATGATACGGCGACCACCGAGATCTACACTCTATGGAGTCGTCGGCAGCGTC |
| 2 | C9 | I5B163 | GGCTGTATA | AATGATACGGCGACCACCGAGATCTACACGGCTGTATATCGTCGGCAGCGTC |
| 2 | D9 | I5B164 | CGCCTAGAA | AATGATACGGCGACCACCGAGATCTACACCGCCTAGAATCGTCGGCAGCGTC |
| 2 | E9 | I5B165 | GATCGGTTA | AATGATACGGCGACCACCGAGATCTACACGATCGGTTATCGTCGGCAGCGTC |
| 2 | F9 | I5B166 | GGCCAATTC | AATGATACGGCGACCACCGAGATCTACACGGCCAATTCTCGTCGGCAGCGTC |
| 2 | G9 | I5B167 | ACAAGGTTC | AATGATACGGCGACCACCGAGATCTACACACAAGGTTCTCGTCGGCAGCGTC |
| 2 | H9 | I5B168 | TCTCCGGAA | AATGATACGGCGACCACCGAGATCTACACTCTCCGGAATCGTCGGCAGCGTC |
| 2 | A10 | I5B169 | CTGTAGTGG | AATGATACGGCGACCACCGAGATCTACACCTGTAGTGGTCGTCGGCAGCGTC |
| 2 | B10 | I5B170 | GTTGGATTG | AATGATACGGCGACCACCGAGATCTACACGTTGGATTGTCGTCGGCAGCGTC |
| 2 | C10 | I5B171 | AGCACCTTG | AATGATACGGCGACCACCGAGATCTACACAGCACCTTGTCGTCGGCAGCGTC |
| 2 | D10 | I5B172 | TCTTAAGCG | AATGATACGGCGACCACCGAGATCTACACTCTTAAGCGTCGTCGGCAGCGTC |
| 2 | E10 | I5B173 | TGGTTATCG | AATGATACGGCGACCACCGAGATCTACACTGGTTATCGTCGTCGGCAGCGTC |
| 2 | F10 | I5B174 | GCAAGCTAA | AATGATACGGCGACCACCGAGATCTACACGCAAGCTAATCGTCGGCAGCGTC |
| 2 | G10 | I5B175 | AACCACTGG | AATGATACGGCGACCACCGAGATCTACACAACCACTGGTCGTCGGCAGCGTC |
| 2 | H10 | I5B176 | TAATGACGG | AATGATACGGCGACCACCGAGATCTACACTAATGACGGTCGTCGGCAGCGTC |
| 2 | A11 | I5B177 | GGTGAGTAA | AATGATACGGCGACCACCGAGATCTACACGGTGAGTAATCGTCGGCAGCGTC |
| 2 | B11 | I5B178 | CGAAGAGAA | AATGATACGGCGACCACCGAGATCTACACCGAAGAGAATCGTCGGCAGCGTC |
| 2 | C11 | I5B179 | ATAACCGGA | AATGATACGGCGACCACCGAGATCTACACATAACCGGATCGTCGGCAGCGTC |
| 2 | D11 | I5B180 | CGCTCATTA | AATGATACGGCGACCACCGAGATCTACACCGCTCATTATCGTCGGCAGCGTC |
| 2 | E11 | I5B181 | TCTGTCTAG | AATGATACGGCGACCACCGAGATCTACACTCTGTCTAGTCGTCGGCAGCGTC |
| 2 | F11 | I5B182 | GAGGAATGG | AATGATACGGCGACCACCGAGATCTACACGAGGAATGGTCGTCGGCAGCGTC |
| 2 | G11 | I5B183 | GCCATACAA | AATGATACGGCGACCACCGAGATCTACACGCCATACAATCGTCGGCAGCGTC |
| 2 | H11 | I5B184 | GGATTGTCG | AATGATACGGCGACCACCGAGATCTACACGGATTGTCGTCGTCGGCAGCGTC |
| 2 | A12 | I5B185 | CCATTCTGT | AATGATACGGCGACCACCGAGATCTACACCCATTCTGTTCGTCGGCAGCGTC |
| 2 | B12 | I5B186 | GCCTAACTA | AATGATACGGCGACCACCGAGATCTACACGCCTAACTATCGTCGGCAGCGTC |
| 2 | C12 | I5B187 | CTCTTCAGA | AATGATACGGCGACCACCGAGATCTACACCTCTTCAGATCGTCGGCAGCGTC |
| 2 | D12 | I5B188 | CACCTACTT | AATGATACGGCGACCACCGAGATCTACACCACCTACTTTCGTCGGCAGCGTC |
| 2 | E12 | I5B189 | GGAAGATTG | AATGATACGGCGACCACCGAGATCTACACGGAAGATTGTCGTCGGCAGCGTC |
| 2 | F12 | I5B190 | GTATCGTTG | AATGATACGGCGACCACCGAGATCTACACGTATCGTTGTCGTCGGCAGCGTC |
| 2 | G12 | I5B191 | TAGCTCTTC | AATGATACGGCGACCACCGAGATCTACACTAGCTCTTCTCGTCGGCAGCGTC |
| 2 | H12 | I5B192 | ACACTCCTA | AATGATACGGCGACCACCGAGATCTACACACACTCCTATCGTCGGCAGCGTC |
| 3 | A1 | I5C193 | CCACAAGAA | AATGATACGGCGACCACCGAGATCTACACCCACAAGAATCGTCGGCAGCGTC |
| 3 | B1 | I5C194 | TGTTGAGGA | AATGATACGGCGACCACCGAGATCTACACTGTTGAGGATCGTCGGCAGCGTC |
| 3 | C1 | I5C195 | AAGGATGCA | AATGATACGGCGACCACCGAGATCTACACAAGGATGCATCGTCGGCAGCGTC |
| 3 | D1 | I5C196 | CACACTCAA | AATGATACGGCGACCACCGAGATCTACACCACACTCAATCGTCGGCAGCGTC |
| 3 | E1 | I5C197 | AGGTTAACC | AATGATACGGCGACCACCGAGATCTACACAGGTTAACCTCGTCGGCAGCGTC |
| 3 | F1 | I5C198 | CCATAACCA | AATGATACGGCGACCACCGAGATCTACACCCATAACCATCGTCGGCAGCGTC |
| 3 | G1 | I5C199 | AAGCCGGTT | AATGATACGGCGACCACCGAGATCTACACAAGCCGGTTTCGTCGGCAGCGTC |
| 3 | H1 | I5C200 | AGCTCCACA | AATGATACGGCGACCACCGAGATCTACACAGCTCCACATCGTCGGCAGCGTC |
| 3 | A2 | I5C201 | CTTGCCGTA | AATGATACGGCGACCACCGAGATCTACACCTTGCCGTATCGTCGGCAGCGTC |
| 3 | B2 | I5C202 | TTAGGCCGA | AATGATACGGCGACCACCGAGATCTACACTTAGGCCGATCGTCGGCAGCGTC |
| 3 | C2 | I5C203 | AGCAAGGAA | AATGATACGGCGACCACCGAGATCTACACAGCAAGGAATCGTCGGCAGCGTC |
| 3 | D2 | I5C204 | GCCTATTGT | AATGATACGGCGACCACCGAGATCTACACGCCTATTGTTCGTCGGCAGCGTC |
| 3 | E2 | I5C205 | ACTGTCCTG | AATGATACGGCGACCACCGAGATCTACACACTGTCCTGTCGTCGGCAGCGTC |
| 3 | F2 | I5C206 | TTAACACCG | AATGATACGGCGACCACCGAGATCTACACTTAACACCGTCGTCGGCAGCGTC |
| 3 | G2 | I5C207 | GGAATCCAA | AATGATACGGCGACCACCGAGATCTACACGGAATCCAATCGTCGGCAGCGTC |
| 3 | H2 | I5C208 | TCCAGGCTT | AATGATACGGCGACCACCGAGATCTACACTCCAGGCTTTCGTCGGCAGCGTC |
| 3 | A3 | I5C209 | CAGAAGGTA | AATGATACGGCGACCACCGAGATCTACACCAGAAGGTATCGTCGGCAGCGTC |
| 3 | B3 | I5C210 | CCAATGCAA | AATGATACGGCGACCACCGAGATCTACACCCAATGCAATCGTCGGCAGCGTC |
| 3 | C3 | I5C211 | AACCTTACC | AATGATACGGCGACCACCGAGATCTACACAACCTTACCTCGTCGGCAGCGTC |
| 3 | D3 | I5C212 | GATGAGTGT | AATGATACGGCGACCACCGAGATCTACACGATGAGTGTTCGTCGGCAGCGTC |
| 3 | E3 | I5C213 | ACCACCGAA | AATGATACGGCGACCACCGAGATCTACACACCACCGAATCGTCGGCAGCGTC |
| 3 | F3 | I5C214 | CTCTGACTC | AATGATACGGCGACCACCGAGATCTACACCTCTGACTCTCGTCGGCAGCGTC |
| 3 | G3 | I5C215 | AATGGCCGA | AATGATACGGCGACCACCGAGATCTACACAATGGCCGATCGTCGGCAGCGTC |
| 3 | H3 | I5C216 | CTGCATCTT | AATGATACGGCGACCACCGAGATCTACACCTGCATCTTTCGTCGGCAGCGTC |
| 3 | A4 | I5C217 | GGACAATGG | AATGATACGGCGACCACCGAGATCTACACGGACAATGGTCGTCGGCAGCGTC |
| 3 | B4 | I5C218 | TTCGGATTC | AATGATACGGCGACCACCGAGATCTACACTTCGGATTCTCGTCGGCAGCGTC |
| 3 | C4 | I5C219 | CAAGGCGAA | AATGATACGGCGACCACCGAGATCTACACCAAGGCGAATCGTCGGCAGCGTC |
| 3 | D4 | I5C220 | CGCACCTAA | AATGATACGGCGACCACCGAGATCTACACCGCACCTAATCGTCGGCAGCGTC |
| 3 | E4 | I5C221 | AGTTACGCC | AATGATACGGCGACCACCGAGATCTACACAGTTACGCCTCGTCGGCAGCGTC |
| 3 | F4 | I5C222 | AACGAAGGC | AATGATACGGCGACCACCGAGATCTACACAACGAAGGCTCGTCGGCAGCGTC |
| 3 | G4 | I5C223 | TGTTCGCTA | AATGATACGGCGACCACCGAGATCTACACTGTTCGCTATCGTCGGCAGCGTC |
| 3 | H4 | I5C224 | CATTCTCCG | AATGATACGGCGACCACCGAGATCTACACCATTCTCCGTCGTCGGCAGCGTC |
| 3 | A5 | I5C225 | TCCGTGTTA | AATGATACGGCGACCACCGAGATCTACACTCCGTGTTATCGTCGGCAGCGTC |
| 3 | B5 | I5C226 | AACAGCAGA | AATGATACGGCGACCACCGAGATCTACACAACAGCAGATCGTCGGCAGCGTC |
| 3 | C5 | I5C227 | TACAAGCGG | AATGATACGGCGACCACCGAGATCTACACTACAAGCGGTCGTCGGCAGCGTC |
| 3 | D5 | I5C228 | GGTGAACTG | AATGATACGGCGACCACCGAGATCTACACGGTGAACTGTCGTCGGCAGCGTC |
| 3 | E5 | I5C229 | CAACTGTTC | AATGATACGGCGACCACCGAGATCTACACCAACTGTTCTCGTCGGCAGCGTC |
| 3 | F5 | I5C230 | ACCAACTCA | AATGATACGGCGACCACCGAGATCTACACACCAACTCATCGTCGGCAGCGTC |
| 3 | G5 | I5C231 | ATCATCACC | AATGATACGGCGACCACCGAGATCTACACATCATCACCTCGTCGGCAGCGTC |
| 3 | H5 | I5C232 | CCTAAGGAA | AATGATACGGCGACCACCGAGATCTACACCCTAAGGAATCGTCGGCAGCGTC |
| 3 | A6 | I5C233 | CCTTGATCC | AATGATACGGCGACCACCGAGATCTACACCCTTGATCCTCGTCGGCAGCGTC |
| 3 | B6 | I5C234 | AGAGACTGA | AATGATACGGCGACCACCGAGATCTACACAGAGACTGATCGTCGGCAGCGTC |
| 3 | C6 | I5C235 | GTGTTACGG | AATGATACGGCGACCACCGAGATCTACACGTGTTACGGTCGTCGGCAGCGTC |
| 3 | D6 | I5C236 | CCTTGTGGA | AATGATACGGCGACCACCGAGATCTACACCCTTGTGGATCGTCGGCAGCGTC |
| 3 | E6 | I5C237 | TTAGAACGG | AATGATACGGCGACCACCGAGATCTACACTTAGAACGGTCGTCGGCAGCGTC |
| 3 | F6 | I5C238 | TCAGAGGAA | AATGATACGGCGACCACCGAGATCTACACTCAGAGGAATCGTCGGCAGCGTC |
| 3 | G6 | I5C239 | AGAAGAGCC | AATGATACGGCGACCACCGAGATCTACACAGAAGAGCCTCGTCGGCAGCGTC |
| 3 | H6 | I5C240 | GCTTACCAA | AATGATACGGCGACCACCGAGATCTACACGCTTACCAATCGTCGGCAGCGTC |
| 3 | A7 | I5C241 | ACCGAATCC | AATGATACGGCGACCACCGAGATCTACACACCGAATCCTCGTCGGCAGCGTC |
| 3 | B7 | I5C242 | TGATAGGCG | AATGATACGGCGACCACCGAGATCTACACTGATAGGCGTCGTCGGCAGCGTC |
| 3 | C7 | I5C243 | CCGTGATGT | AATGATACGGCGACCACCGAGATCTACACCCGTGATGTTCGTCGGCAGCGTC |
| 3 | D7 | I5C244 | CAGCCTTAA | AATGATACGGCGACCACCGAGATCTACACCAGCCTTAATCGTCGGCAGCGTC |
| 3 | E7 | I5C245 | CACCAAGCA | AATGATACGGCGACCACCGAGATCTACACCACCAAGCATCGTCGGCAGCGTC |
| 3 | F7 | I5C246 | TATCGCCAA | AATGATACGGCGACCACCGAGATCTACACTATCGCCAATCGTCGGCAGCGTC |
| 3 | G7 | I5C247 | AACTGTGGA | AATGATACGGCGACCACCGAGATCTACACAACTGTGGATCGTCGGCAGCGTC |
| 3 | H7 | I5C248 | TCTGCGTTG | AATGATACGGCGACCACCGAGATCTACACTCTGCGTTGTCGTCGGCAGCGTC |
| 3 | A8 | I5C249 | CAATCCTCA | AATGATACGGCGACCACCGAGATCTACACCAATCCTCATCGTCGGCAGCGTC |
| 3 | B8 | I5C250 | CATACCGCA | AATGATACGGCGACCACCGAGATCTACACCATACCGCATCGTCGGCAGCGTC |
| 3 | C8 | I5C251 | TAAGCGTCC | AATGATACGGCGACCACCGAGATCTACACTAAGCGTCCTCGTCGGCAGCGTC |
| 3 | D8 | I5C252 | CGGAGTGTA | AATGATACGGCGACCACCGAGATCTACACCGGAGTGTATCGTCGGCAGCGTC |
| 3 | E8 | I5C253 | TCATGTTCG | AATGATACGGCGACCACCGAGATCTACACTCATGTTCGTCGTCGGCAGCGTC |
| 3 | F8 | I5C254 | GGAACGTGT | AATGATACGGCGACCACCGAGATCTACACGGAACGTGTTCGTCGGCAGCGTC |
| 3 | G8 | I5C255 | GTTCCGCAA | AATGATACGGCGACCACCGAGATCTACACGTTCCGCAATCGTCGGCAGCGTC |
| 3 | H8 | I5C256 | CCGAGATAA | AATGATACGGCGACCACCGAGATCTACACCCGAGATAATCGTCGGCAGCGTC |
| 3 | A9 | I5C257 | AGGAAGTCA | AATGATACGGCGACCACCGAGATCTACACAGGAAGTCATCGTCGGCAGCGTC |
| 3 | B9 | I5C258 | GTTGGCCAA | AATGATACGGCGACCACCGAGATCTACACGTTGGCCAATCGTCGGCAGCGTC |
| 3 | C9 | I5C259 | TATGGTGGA | AATGATACGGCGACCACCGAGATCTACACTATGGTGGATCGTCGGCAGCGTC |
| 3 | D9 | I5C260 | CTATTGCGG | AATGATACGGCGACCACCGAGATCTACACCTATTGCGGTCGTCGGCAGCGTC |
| 3 | E9 | I5C261 | CAGTTCCTG | AATGATACGGCGACCACCGAGATCTACACCAGTTCCTGTCGTCGGCAGCGTC |
| 3 | F9 | I5C262 | TGGCAAGAA | AATGATACGGCGACCACCGAGATCTACACTGGCAAGAATCGTCGGCAGCGTC |
| 3 | G9 | I5C263 | TACCTCGCT | AATGATACGGCGACCACCGAGATCTACACTACCTCGCTTCGTCGGCAGCGTC |
| 3 | H9 | I5C264 | ATAGGAGGA | AATGATACGGCGACCACCGAGATCTACACATAGGAGGATCGTCGGCAGCGTC |
| 3 | A10 | I5C265 | CGACAGTAA | AATGATACGGCGACCACCGAGATCTACACCGACAGTAATCGTCGGCAGCGTC |
| 3 | B10 | I5C266 | TGTGGCTTA | AATGATACGGCGACCACCGAGATCTACACTGTGGCTTATCGTCGGCAGCGTC |
| 3 | C10 | I5C267 | TTCTGTCGG | AATGATACGGCGACCACCGAGATCTACACTTCTGTCGGTCGTCGGCAGCGTC |
| 3 | D10 | I5C268 | TGGAGCTAG | AATGATACGGCGACCACCGAGATCTACACTGGAGCTAGTCGTCGGCAGCGTC |
| 3 | E10 | I5C269 | TTAGTGAGC | AATGATACGGCGACCACCGAGATCTACACTTAGTGAGCTCGTCGGCAGCGTC |
| 3 | F10 | I5C270 | ATGAGGCAA | AATGATACGGCGACCACCGAGATCTACACATGAGGCAATCGTCGGCAGCGTC |
| 3 | G10 | I5C271 | CTCTCCTTG | AATGATACGGCGACCACCGAGATCTACACCTCTCCTTGTCGTCGGCAGCGTC |
| 3 | H10 | I5C272 | AGAAGCTCG | AATGATACGGCGACCACCGAGATCTACACAGAAGCTCGTCGTCGGCAGCGTC |
| 3 | A11 | I5C273 | TGACAACCA | AATGATACGGCGACCACCGAGATCTACACTGACAACCATCGTCGGCAGCGTC |
| 3 | B11 | I5C274 | ATTGGTACG | AATGATACGGCGACCACCGAGATCTACACATTGGTACGTCGTCGGCAGCGTC |
| 3 | C11 | I5C275 | AGCGCGTTA | AATGATACGGCGACCACCGAGATCTACACAGCGCGTTATCGTCGGCAGCGTC |
| 3 | D11 | I5C276 | CAGGAAGAA | AATGATACGGCGACCACCGAGATCTACACCAGGAAGAATCGTCGGCAGCGTC |
| 3 | E11 | I5C277 | CCATATTGG | AATGATACGGCGACCACCGAGATCTACACCCATATTGGTCGTCGGCAGCGTC |
| 3 | F11 | I5C278 | TGCCGTTCT | AATGATACGGCGACCACCGAGATCTACACTGCCGTTCTTCGTCGGCAGCGTC |
| 3 | G11 | I5C279 | AAGGTCGGA | AATGATACGGCGACCACCGAGATCTACACAAGGTCGGATCGTCGGCAGCGTC |
| 3 | H11 | I5C280 | GAGAGACAA | AATGATACGGCGACCACCGAGATCTACACGAGAGACAATCGTCGGCAGCGTC |
| 3 | A12 | I5C281 | CGGTTAGTT | AATGATACGGCGACCACCGAGATCTACACCGGTTAGTTTCGTCGGCAGCGTC |
| 3 | B12 | I5C282 | GTGGAACAA | AATGATACGGCGACCACCGAGATCTACACGTGGAACAATCGTCGGCAGCGTC |
| 3 | C12 | I5C283 | GCCAACGTT | AATGATACGGCGACCACCGAGATCTACACGCCAACGTTTCGTCGGCAGCGTC |
| 3 | D12 | I5C284 | GAACACCAA | AATGATACGGCGACCACCGAGATCTACACGAACACCAATCGTCGGCAGCGTC |
| 3 | E12 | I5C285 | CGGATATGA | AATGATACGGCGACCACCGAGATCTACACCGGATATGATCGTCGGCAGCGTC |
| 3 | F12 | I5C286 | TATGAGACG | AATGATACGGCGACCACCGAGATCTACACTATGAGACGTCGTCGGCAGCGTC |
| 3 | G12 | I5C287 | TGTCCTCGA | AATGATACGGCGACCACCGAGATCTACACTGTCCTCGATCGTCGGCAGCGTC |
| 3 | H12 | I5C288 | ATACAGGTG | AATGATACGGCGACCACCGAGATCTACACATACAGGTGTCGTCGGCAGCGTC |
| 4 | A1 | I5D289 | CCACTTACA | AATGATACGGCGACCACCGAGATCTACACCCACTTACATCGTCGGCAGCGTC |
| 4 | B1 | I5D290 | CAACAACGG | AATGATACGGCGACCACCGAGATCTACACCAACAACGGTCGTCGGCAGCGTC |
| 4 | C1 | I5D291 | AATCCAGCC | AATGATACGGCGACCACCGAGATCTACACAATCCAGCCTCGTCGGCAGCGTC |
| 4 | D1 | I5D292 | GGATAGCAA | AATGATACGGCGACCACCGAGATCTACACGGATAGCAATCGTCGGCAGCGTC |
| 4 | E1 | I5D293 | GAACTTCGG | AATGATACGGCGACCACCGAGATCTACACGAACTTCGGTCGTCGGCAGCGTC |
| 4 | F1 | I5D294 | GTGGAAGTT | AATGATACGGCGACCACCGAGATCTACACGTGGAAGTTTCGTCGGCAGCGTC |
| 4 | G1 | I5D295 | TGTCTTGCC | AATGATACGGCGACCACCGAGATCTACACTGTCTTGCCTCGTCGGCAGCGTC |
| 4 | H1 | I5D296 | GAAGTGGAA | AATGATACGGCGACCACCGAGATCTACACGAAGTGGAATCGTCGGCAGCGTC |
| 4 | A2 | I5D297 | TCCGTCCAA | AATGATACGGCGACCACCGAGATCTACACTCCGTCCAATCGTCGGCAGCGTC |
| 4 | B2 | I5D298 | GTGGTCGAA | AATGATACGGCGACCACCGAGATCTACACGTGGTCGAATCGTCGGCAGCGTC |
| 4 | C2 | I5D299 | GCCTTGGAA | AATGATACGGCGACCACCGAGATCTACACGCCTTGGAATCGTCGGCAGCGTC |
| 4 | D2 | I5D300 | GGAGAAGAA | AATGATACGGCGACCACCGAGATCTACACGGAGAAGAATCGTCGGCAGCGTC |
| 4 | E2 | I5D301 | TTGCACCAA | AATGATACGGCGACCACCGAGATCTACACTTGCACCAATCGTCGGCAGCGTC |
| 4 | F2 | I5D302 | CCAAGATGG | AATGATACGGCGACCACCGAGATCTACACCCAAGATGGTCGTCGGCAGCGTC |
| 4 | G2 | I5D303 | ATCCGCTCA | AATGATACGGCGACCACCGAGATCTACACATCCGCTCATCGTCGGCAGCGTC |
| 4 | H2 | I5D304 | CTTGAATGG | AATGATACGGCGACCACCGAGATCTACACCTTGAATGGTCGTCGGCAGCGTC |
| 4 | A3 | I5D305 | CTGGTAGGA | AATGATACGGCGACCACCGAGATCTACACCTGGTAGGATCGTCGGCAGCGTC |
| 4 | B3 | I5D306 | GGAGCCTAA | AATGATACGGCGACCACCGAGATCTACACGGAGCCTAATCGTCGGCAGCGTC |
| 4 | C3 | I5D307 | CGTAGGTTG | AATGATACGGCGACCACCGAGATCTACACCGTAGGTTGTCGTCGGCAGCGTC |
| 4 | D3 | I5D308 | CATAAGTGG | AATGATACGGCGACCACCGAGATCTACACCATAAGTGGTCGTCGGCAGCGTC |
| 4 | E3 | I5D309 | CATCTGCTG | AATGATACGGCGACCACCGAGATCTACACCATCTGCTGTCGTCGGCAGCGTC |
| 4 | F3 | I5D310 | TCAAGACCA | AATGATACGGCGACCACCGAGATCTACACTCAAGACCATCGTCGGCAGCGTC |
| 4 | G3 | I5D311 | TTGCATGTG | AATGATACGGCGACCACCGAGATCTACACTTGCATGTGTCGTCGGCAGCGTC |
| 4 | H3 | I5D312 | TTCTCACGA | AATGATACGGCGACCACCGAGATCTACACTTCTCACGATCGTCGGCAGCGTC |
| 4 | A4 | I5D313 | CAATAGAGG | AATGATACGGCGACCACCGAGATCTACACCAATAGAGGTCGTCGGCAGCGTC |
| 4 | B4 | I5D314 | ACCTGCCAA | AATGATACGGCGACCACCGAGATCTACACACCTGCCAATCGTCGGCAGCGTC |
| 4 | C4 | I5D315 | CTACCATCA | AATGATACGGCGACCACCGAGATCTACACCTACCATCATCGTCGGCAGCGTC |
| 4 | D4 | I5D316 | TTCCTTCCG | AATGATACGGCGACCACCGAGATCTACACTTCCTTCCGTCGTCGGCAGCGTC |
| 4 | E4 | I5D317 | AACGAGTGA | AATGATACGGCGACCACCGAGATCTACACAACGAGTGATCGTCGGCAGCGTC |
| 4 | F4 | I5D318 | AACATTGCG | AATGATACGGCGACCACCGAGATCTACACAACATTGCGTCGTCGGCAGCGTC |
| 4 | G4 | I5D319 | CCTCTCGAA | AATGATACGGCGACCACCGAGATCTACACCCTCTCGAATCGTCGGCAGCGTC |
| 4 | H4 | I5D320 | TCTGGCGAA | AATGATACGGCGACCACCGAGATCTACACTCTGGCGAATCGTCGGCAGCGTC |
| 4 | A5 | I5D321 | CCTGAACAA | AATGATACGGCGACCACCGAGATCTACACCCTGAACAATCGTCGGCAGCGTC |
| 4 | B5 | I5D322 | TCGGTAGAA | AATGATACGGCGACCACCGAGATCTACACTCGGTAGAATCGTCGGCAGCGTC |
| 4 | C5 | I5D323 | GTTGTGGTA | AATGATACGGCGACCACCGAGATCTACACGTTGTGGTATCGTCGGCAGCGTC |
| 4 | D5 | I5D324 | AATGTCACC | AATGATACGGCGACCACCGAGATCTACACAATGTCACCTCGTCGGCAGCGTC |
| 4 | E5 | I5D325 | AGTGAGTTG | AATGATACGGCGACCACCGAGATCTACACAGTGAGTTGTCGTCGGCAGCGTC |
| 4 | F5 | I5D326 | CGGCTTCTA | AATGATACGGCGACCACCGAGATCTACACCGGCTTCTATCGTCGGCAGCGTC |
| 4 | G5 | I5D327 | TTATCCGCG | AATGATACGGCGACCACCGAGATCTACACTTATCCGCGTCGTCGGCAGCGTC |
| 4 | H5 | I5D328 | AGGTGACAA | AATGATACGGCGACCACCGAGATCTACACAGGTGACAATCGTCGGCAGCGTC |
| 4 | A6 | I5D329 | TTCTCCTCA | AATGATACGGCGACCACCGAGATCTACACTTCTCCTCATCGTCGGCAGCGTC |
| 4 | B6 | I5D330 | CGGAACTCT | AATGATACGGCGACCACCGAGATCTACACCGGAACTCTTCGTCGGCAGCGTC |
| 4 | C6 | I5D331 | AGAACGCAA | AATGATACGGCGACCACCGAGATCTACACAGAACGCAATCGTCGGCAGCGTC |
| 4 | D6 | I5D332 | TCCTCTACA | AATGATACGGCGACCACCGAGATCTACACTCCTCTACATCGTCGGCAGCGTC |
| 4 | E6 | I5D333 | CAAGGAGTC | AATGATACGGCGACCACCGAGATCTACACCAAGGAGTCTCGTCGGCAGCGTC |
| 4 | F6 | I5D334 | GGATGTTCA | AATGATACGGCGACCACCGAGATCTACACGGATGTTCATCGTCGGCAGCGTC |
| 4 | G6 | I5D335 | AGAGGTCCA | AATGATACGGCGACCACCGAGATCTACACAGAGGTCCATCGTCGGCAGCGTC |
| 4 | H6 | I5D336 | CCAACCTGA | AATGATACGGCGACCACCGAGATCTACACCCAACCTGATCGTCGGCAGCGTC |
| 4 | A7 | I5D337 | AAGACAGGA | AATGATACGGCGACCACCGAGATCTACACAAGACAGGATCGTCGGCAGCGTC |
| 4 | B7 | I5D338 | CGAATATCC | AATGATACGGCGACCACCGAGATCTACACCGAATATCCTCGTCGGCAGCGTC |
| 4 | C7 | I5D339 | CCTAGTTCG | AATGATACGGCGACCACCGAGATCTACACCCTAGTTCGTCGTCGGCAGCGTC |
| 4 | D7 | I5D340 | AGTGGAGAA | AATGATACGGCGACCACCGAGATCTACACAGTGGAGAATCGTCGGCAGCGTC |
| 4 | E7 | I5D341 | GGCAGGTAA | AATGATACGGCGACCACCGAGATCTACACGGCAGGTAATCGTCGGCAGCGTC |
| 4 | F7 | I5D342 | TCACCACAA | AATGATACGGCGACCACCGAGATCTACACTCACCACAATCGTCGGCAGCGTC |
| 4 | G7 | I5D343 | TAGGCACAA | AATGATACGGCGACCACCGAGATCTACACTAGGCACAATCGTCGGCAGCGTC |
| 4 | H7 | I5D344 | TATTGCTCG | AATGATACGGCGACCACCGAGATCTACACTATTGCTCGTCGTCGGCAGCGTC |
| 4 | A8 | I5D345 | CAGATCCAA | AATGATACGGCGACCACCGAGATCTACACCAGATCCAATCGTCGGCAGCGTC |
| 4 | B8 | I5D346 | TCGAACGAA | AATGATACGGCGACCACCGAGATCTACACTCGAACGAATCGTCGGCAGCGTC |
| 4 | C8 | I5D347 | TTCCAACAG | AATGATACGGCGACCACCGAGATCTACACTTCCAACAGTCGTCGGCAGCGTC |
| 4 | D8 | I5D348 | ACCGAAGAA | AATGATACGGCGACCACCGAGATCTACACACCGAAGAATCGTCGGCAGCGTC |
| 4 | E8 | I5D349 | GACCTTCAA | AATGATACGGCGACCACCGAGATCTACACGACCTTCAATCGTCGGCAGCGTC |
| 4 | F8 | I5D350 | ATGCTGCTT | AATGATACGGCGACCACCGAGATCTACACATGCTGCTTTCGTCGGCAGCGTC |
| 4 | G8 | I5D351 | CCGGAATTA | AATGATACGGCGACCACCGAGATCTACACCCGGAATTATCGTCGGCAGCGTC |
| 4 | H8 | I5D352 | CACAACTAG | AATGATACGGCGACCACCGAGATCTACACCACAACTAGTCGTCGGCAGCGTC |
| 4 | A9 | I5D353 | GTGCGTATG | AATGATACGGCGACCACCGAGATCTACACGTGCGTATGTCGTCGGCAGCGTC |
| 4 | B9 | I5D354 | GGCTTGGTT | AATGATACGGCGACCACCGAGATCTACACGGCTTGGTTTCGTCGGCAGCGTC |
| 4 | C9 | I5D355 | AAGAACCGA | AATGATACGGCGACCACCGAGATCTACACAAGAACCGATCGTCGGCAGCGTC |
| 4 | D9 | I5D356 | AAGGTGTTG | AATGATACGGCGACCACCGAGATCTACACAAGGTGTTGTCGTCGGCAGCGTC |
| 4 | E9 | I5D357 | GCGTAAGAA | AATGATACGGCGACCACCGAGATCTACACGCGTAAGAATCGTCGGCAGCGTC |
| 4 | F9 | I5D358 | CAACGACAA | AATGATACGGCGACCACCGAGATCTACACCAACGACAATCGTCGGCAGCGTC |
| 4 | G9 | I5D359 | GGTCCTTAA | AATGATACGGCGACCACCGAGATCTACACGGTCCTTAATCGTCGGCAGCGTC |
| 4 | H9 | I5D360 | AGAACATGG | AATGATACGGCGACCACCGAGATCTACACAGAACATGGTCGTCGGCAGCGTC |
| 4 | A10 | I5D361 | TCGTGTGCA | AATGATACGGCGACCACCGAGATCTACACTCGTGTGCATCGTCGGCAGCGTC |
| 4 | B10 | I5D362 | AACACGTTC | AATGATACGGCGACCACCGAGATCTACACAACACGTTCTCGTCGGCAGCGTC |
| 4 | C10 | I5D363 | AACCGGCAA | AATGATACGGCGACCACCGAGATCTACACAACCGGCAATCGTCGGCAGCGTC |
| 4 | D10 | I5D364 | TTCCTGGTA | AATGATACGGCGACCACCGAGATCTACACTTCCTGGTATCGTCGGCAGCGTC |
| 4 | E10 | I5D365 | GACGGAGAA | AATGATACGGCGACCACCGAGATCTACACGACGGAGAATCGTCGGCAGCGTC |
| 4 | F10 | I5D366 | CGTCACTTC | AATGATACGGCGACCACCGAGATCTACACCGTCACTTCTCGTCGGCAGCGTC |
| 4 | G10 | I5D367 | TTCCGCCTA | AATGATACGGCGACCACCGAGATCTACACTTCCGCCTATCGTCGGCAGCGTC |
| 4 | H10 | I5D368 | GGACGATAA | AATGATACGGCGACCACCGAGATCTACACGGACGATAATCGTCGGCAGCGTC |
| 4 | A11 | I5D369 | CGGCAATCA | AATGATACGGCGACCACCGAGATCTACACCGGCAATCATCGTCGGCAGCGTC |
| 4 | B11 | I5D370 | TCCTCTTGG | AATGATACGGCGACCACCGAGATCTACACTCCTCTTGGTCGTCGGCAGCGTC |
| 4 | C11 | I5D371 | GGTGCATTC | AATGATACGGCGACCACCGAGATCTACACGGTGCATTCTCGTCGGCAGCGTC |
| 4 | D11 | I5D372 | GGCCACTAA | AATGATACGGCGACCACCGAGATCTACACGGCCACTAATCGTCGGCAGCGTC |
| 4 | E11 | I5D373 | GAAGGACTT | AATGATACGGCGACCACCGAGATCTACACGAAGGACTTTCGTCGGCAGCGTC |
| 4 | F11 | I5D374 | TTCGAGTGG | AATGATACGGCGACCACCGAGATCTACACTTCGAGTGGTCGTCGGCAGCGTC |
| 4 | G11 | I5D375 | GGTATGGAA | AATGATACGGCGACCACCGAGATCTACACGGTATGGAATCGTCGGCAGCGTC |
| 4 | H11 | I5D376 | GTCTTCTGG | AATGATACGGCGACCACCGAGATCTACACGTCTTCTGGTCGTCGGCAGCGTC |
| 4 | A12 | I5D377 | CCTCGGTAA | AATGATACGGCGACCACCGAGATCTACACCCTCGGTAATCGTCGGCAGCGTC |
| 4 | B12 | I5D378 | TTGCCTGAA | AATGATACGGCGACCACCGAGATCTACACTTGCCTGAATCGTCGGCAGCGTC |
| 4 | C12 | I5D379 | ACCAGTTGG | AATGATACGGCGACCACCGAGATCTACACACCAGTTGGTCGTCGGCAGCGTC |
| 4 | D12 | I5D380 | GACAGGTTG | AATGATACGGCGACCACCGAGATCTACACGACAGGTTGTCGTCGGCAGCGTC |
| 4 | E12 | I5D381 | AGCGACCAA | AATGATACGGCGACCACCGAGATCTACACAGCGACCAATCGTCGGCAGCGTC |
| 4 | F12 | I5D382 | TTGTACCGG | AATGATACGGCGACCACCGAGATCTACACTTGTACCGGTCGTCGGCAGCGTC |
| 4 | G12 | I5D383 | ACTGGTGTG | AATGATACGGCGACCACCGAGATCTACACACTGGTGTGTCGTCGGCAGCGTC |
| 4 | H12 | I5D384 | CCACGACTT | AATGATACGGCGACCACCGAGATCTACACCCACGACTTTCGTCGGCAGCGTC |
| 1 | A1 | I7A001 | CAAGTGAGC | CAAGCAGAAGACGGCATACGAGATGCTCACTTGGTCTCGTGGGCTCGG |
| 1 | B1 | I7A002 | GAACTCCGT | CAAGCAGAAGACGGCATACGAGATACGGAGTTCGTCTCGTGGGCTCGG |
| 1 | C1 | I7A003 | CAGACTAAG | CAAGCAGAAGACGGCATACGAGATCTTAGTCTGGTCTCGTGGGCTCGG |
| 1 | D1 | I7A004 | CAACCGAAT | CAAGCAGAAGACGGCATACGAGATATTCGGTTGGTCTCGTGGGCTCGG |
| 1 | E1 | I7A005 | TGATACCTC | CAAGCAGAAGACGGCATACGAGATGAGGTATCAGTCTCGTGGGCTCGG |
| 1 | F1 | I7A006 | TATACCGGT | CAAGCAGAAGACGGCATACGAGATACCGGTATAGTCTCGTGGGCTCGG |
| 1 | G1 | I7A007 | CACTATCTC | CAAGCAGAAGACGGCATACGAGATGAGATAGTGGTCTCGTGGGCTCGG |
| 1 | H1 | I7A008 | TGATCTACC | CAAGCAGAAGACGGCATACGAGATGGTAGATCAGTCTCGTGGGCTCGG |
| 1 | A2 | I7A009 | TCTCTCCAA | CAAGCAGAAGACGGCATACGAGATTTGGAGAGAGTCTCGTGGGCTCGG |
| 1 | B2 | I7A010 | TTCGAGGTT | CAAGCAGAAGACGGCATACGAGATAACCTCGAAGTCTCGTGGGCTCGG |
| 1 | C2 | I7A011 | TTCCGTCCA | CAAGCAGAAGACGGCATACGAGATTGGACGGAAGTCTCGTGGGCTCGG |
| 1 | D2 | I7A012 | CGAGACATC | CAAGCAGAAGACGGCATACGAGATGATGTCTCGGTCTCGTGGGCTCGG |
| 1 | E2 | I7A013 | CGAGTATTC | CAAGCAGAAGACGGCATACGAGATGAATACTCGGTCTCGTGGGCTCGG |
| 1 | F2 | I7A014 | TCAGCACAC | CAAGCAGAAGACGGCATACGAGATGTGTGCTGAGTCTCGTGGGCTCGG |
| 1 | G2 | I7A015 | TGGAGAACC | CAAGCAGAAGACGGCATACGAGATGGTTCTCCAGTCTCGTGGGCTCGG |
| 1 | H2 | I7A016 | ACAGACGAT | CAAGCAGAAGACGGCATACGAGATATCGTCTGTGTCTCGTGGGCTCGG |
| 1 | A3 | I7A017 | CTCTTGTTC | CAAGCAGAAGACGGCATACGAGATGAACAAGAGGTCTCGTGGGCTCGG |
| 1 | B3 | I7A018 | TCGAAGAAC | CAAGCAGAAGACGGCATACGAGATGTTCTTCGAGTCTCGTGGGCTCGG |
| 1 | C3 | I7A019 | GGTTGGTAT | CAAGCAGAAGACGGCATACGAGATATACCAACCGTCTCGTGGGCTCGG |
| 1 | D3 | I7A020 | TGGAACACG | CAAGCAGAAGACGGCATACGAGATCGTGTTCCAGTCTCGTGGGCTCGG |
| 1 | E3 | I7A021 | TTAGGAAGG | CAAGCAGAAGACGGCATACGAGATCCTTCCTAAGTCTCGTGGGCTCGG |
| 1 | F3 | I7A022 | TGTGTGAAG | CAAGCAGAAGACGGCATACGAGATCTTCACACAGTCTCGTGGGCTCGG |
| 1 | G3 | I7A023 | CAGATATCC | CAAGCAGAAGACGGCATACGAGATGGATATCTGGTCTCGTGGGCTCGG |
| 1 | H3 | I7A024 | TATAGGCCA | CAAGCAGAAGACGGCATACGAGATTGGCCTATAGTCTCGTGGGCTCGG |
| 1 | A4 | I7A025 | CCTGACAAT | CAAGCAGAAGACGGCATACGAGATATTGTCAGGGTCTCGTGGGCTCGG |
| 1 | B4 | I7A026 | TTCCTTCAC | CAAGCAGAAGACGGCATACGAGATGTGAAGGAAGTCTCGTGGGCTCGG |
| 1 | C4 | I7A027 | AATGGTCGG | CAAGCAGAAGACGGCATACGAGATCCGACCATTGTCTCGTGGGCTCGG |
| 1 | D4 | I7A028 | TATAGACGG | CAAGCAGAAGACGGCATACGAGATCCGTCTATAGTCTCGTGGGCTCGG |
| 1 | E4 | I7A029 | GGTAGAGAG | CAAGCAGAAGACGGCATACGAGATCTCTCTACCGTCTCGTGGGCTCGG |
| 1 | F4 | I7A030 | GAAGACAGG | CAAGCAGAAGACGGCATACGAGATCCTGTCTTCGTCTCGTGGGCTCGG |
| 1 | G4 | I7A031 | CACAAGTCA | CAAGCAGAAGACGGCATACGAGATTGACTTGTGGTCTCGTGGGCTCGG |
| 1 | H4 | I7A032 | GTGTTGCTA | CAAGCAGAAGACGGCATACGAGATTAGCAACACGTCTCGTGGGCTCGG |
| 1 | A5 | I7A033 | TTGTCTGCA | CAAGCAGAAGACGGCATACGAGATTGCAGACAAGTCTCGTGGGCTCGG |
| 1 | B5 | I7A034 | TTGTGAGTC | CAAGCAGAAGACGGCATACGAGATGACTCACAAGTCTCGTGGGCTCGG |
| 1 | C5 | I7A035 | TTCTGGTCT | CAAGCAGAAGACGGCATACGAGATAGACCAGAAGTCTCGTGGGCTCGG |
| 1 | D5 | I7A036 | GCAACAGTA | CAAGCAGAAGACGGCATACGAGATTACTGTTGCGTCTCGTGGGCTCGG |
| 1 | E5 | I7A037 | CCATCACAA | CAAGCAGAAGACGGCATACGAGATTTGTGATGGGTCTCGTGGGCTCGG |
| 1 | F5 | I7A038 | TTCTGAACC | CAAGCAGAAGACGGCATACGAGATGGTTCAGAAGTCTCGTGGGCTCGG |
| 1 | G5 | I7A039 | TGACACCAA | CAAGCAGAAGACGGCATACGAGATTTGGTGTCAGTCTCGTGGGCTCGG |
| 1 | H5 | I7A040 | CGTGTGTAC | CAAGCAGAAGACGGCATACGAGATGTACACACGGTCTCGTGGGCTCGG |
| 1 | A6 | I7A041 | GAAGCAAGT | CAAGCAGAAGACGGCATACGAGATACTTGCTTCGTCTCGTGGGCTCGG |
| 1 | B6 | I7A042 | TGCTGAGGT | CAAGCAGAAGACGGCATACGAGATACCTCAGCAGTCTCGTGGGCTCGG |
| 1 | C6 | I7A043 | TGGAGTTAG | CAAGCAGAAGACGGCATACGAGATCTAACTCCAGTCTCGTGGGCTCGG |
| 1 | D6 | I7A044 | TCCGGAATT | CAAGCAGAAGACGGCATACGAGATAATTCCGGAGTCTCGTGGGCTCGG |
| 1 | E6 | I7A045 | AGAGGCTTC | CAAGCAGAAGACGGCATACGAGATGAAGCCTCTGTCTCGTGGGCTCGG |
| 1 | F6 | I7A046 | AGACCGCAA | CAAGCAGAAGACGGCATACGAGATTTGCGGTCTGTCTCGTGGGCTCGG |
| 1 | G6 | I7A047 | CGTCTACTT | CAAGCAGAAGACGGCATACGAGATAAGTAGACGGTCTCGTGGGCTCGG |
| 1 | H6 | I7A048 | AAGTCAACC | CAAGCAGAAGACGGCATACGAGATGGTTGACTTGTCTCGTGGGCTCGG |
| 1 | A7 | I7A049 | TGTGTGGTT | CAAGCAGAAGACGGCATACGAGATAACCACACAGTCTCGTGGGCTCGG |
| 1 | B7 | I7A050 | CAGAGAAGC | CAAGCAGAAGACGGCATACGAGATGCTTCTCTGGTCTCGTGGGCTCGG |
| 1 | C7 | I7A051 | TTAGAGCGC | CAAGCAGAAGACGGCATACGAGATGCGCTCTAAGTCTCGTGGGCTCGG |
| 1 | D7 | I7A052 | CCGTATTCC | CAAGCAGAAGACGGCATACGAGATGGAATACGGGTCTCGTGGGCTCGG |
| 1 | E7 | I7A053 | TAATCCTCG | CAAGCAGAAGACGGCATACGAGATCGAGGATTAGTCTCGTGGGCTCGG |
| 1 | F7 | I7A054 | CAGATGAGA | CAAGCAGAAGACGGCATACGAGATTCTCATCTGGTCTCGTGGGCTCGG |
| 1 | G7 | I7A055 | TTGTTGCGT | CAAGCAGAAGACGGCATACGAGATACGCAACAAGTCTCGTGGGCTCGG |
| 1 | H7 | I7A056 | CCACGTTAA | CAAGCAGAAGACGGCATACGAGATTTAACGTGGGTCTCGTGGGCTCGG |
| 1 | A8 | I7A057 | TTCCGATTG | CAAGCAGAAGACGGCATACGAGATCAATCGGAAGTCTCGTGGGCTCGG |
| 1 | B8 | I7A058 | CACACATTG | CAAGCAGAAGACGGCATACGAGATCAATGTGTGGTCTCGTGGGCTCGG |
| 1 | C8 | I7A059 | TTATGCGTG | CAAGCAGAAGACGGCATACGAGATCACGCATAAGTCTCGTGGGCTCGG |
| 1 | D8 | I7A060 | TATACCACG | CAAGCAGAAGACGGCATACGAGATCGTGGTATAGTCTCGTGGGCTCGG |
| 1 | E8 | I7A061 | CGGCATTGT | CAAGCAGAAGACGGCATACGAGATACAATGCCGGTCTCGTGGGCTCGG |
| 1 | F8 | I7A062 | TTGCGTGGA | CAAGCAGAAGACGGCATACGAGATTCCACGCAAGTCTCGTGGGCTCGG |
| 1 | G8 | I7A063 | TTCTCACAG | CAAGCAGAAGACGGCATACGAGATCTGTGAGAAGTCTCGTGGGCTCGG |
| 1 | H8 | I7A064 | TACCAACCA | CAAGCAGAAGACGGCATACGAGATTGGTTGGTAGTCTCGTGGGCTCGG |
| 1 | A9 | I7A065 | TGAGGTTCA | CAAGCAGAAGACGGCATACGAGATTGAACCTCAGTCTCGTGGGCTCGG |
| 1 | B9 | I7A066 | GTGTCTGTG | CAAGCAGAAGACGGCATACGAGATCACAGACACGTCTCGTGGGCTCGG |
| 1 | C9 | I7A067 | ACACAGGCT | CAAGCAGAAGACGGCATACGAGATAGCCTGTGTGTCTCGTGGGCTCGG |
| 1 | D9 | I7A068 | CTCCAATAC | CAAGCAGAAGACGGCATACGAGATGTATTGGAGGTCTCGTGGGCTCGG |
| 1 | E9 | I7A069 | GGCTCGTTA | CAAGCAGAAGACGGCATACGAGATTAACGAGCCGTCTCGTGGGCTCGG |
| 1 | F9 | I7A070 | TAAGACGCC | CAAGCAGAAGACGGCATACGAGATGGCGTCTTAGTCTCGTGGGCTCGG |
| 1 | G9 | I7A071 | GAGCACAAT | CAAGCAGAAGACGGCATACGAGATATTGTGCTCGTCTCGTGGGCTCGG |
| 1 | H9 | I7A072 | GAAGCTCTT | CAAGCAGAAGACGGCATACGAGATAAGAGCTTCGTCTCGTGGGCTCGG |
| 1 | A10 | I7A073 | CCTAAGCTT | CAAGCAGAAGACGGCATACGAGATAAGCTTAGGGTCTCGTGGGCTCGG |
| 1 | B10 | I7A074 | TAGGTCGTC | CAAGCAGAAGACGGCATACGAGATGACGACCTAGTCTCGTGGGCTCGG |
| 1 | C10 | I7A075 | TTAATCGGC | CAAGCAGAAGACGGCATACGAGATGCCGATTAAGTCTCGTGGGCTCGG |
| 1 | D10 | I7A076 | TTCCACGCA | CAAGCAGAAGACGGCATACGAGATTGCGTGGAAGTCTCGTGGGCTCGG |
| 1 | E10 | I7A077 | TAGGTGAAC | CAAGCAGAAGACGGCATACGAGATGTTCACCTAGTCTCGTGGGCTCGG |
| 1 | F10 | I7A078 | AAGTGGTCA | CAAGCAGAAGACGGCATACGAGATTGACCACTTGTCTCGTGGGCTCGG |
| 1 | G10 | I7A079 | TTATGCCGC | CAAGCAGAAGACGGCATACGAGATGCGGCATAAGTCTCGTGGGCTCGG |
| 1 | H10 | I7A080 | AACCACGTG | CAAGCAGAAGACGGCATACGAGATCACGTGGTTGTCTCGTGGGCTCGG |
| 1 | A11 | I7A081 | TTGTCCTGT | CAAGCAGAAGACGGCATACGAGATACAGGACAAGTCTCGTGGGCTCGG |
| 1 | B11 | I7A082 | CGAATACCT | CAAGCAGAAGACGGCATACGAGATAGGTATTCGGTCTCGTGGGCTCGG |
| 1 | C11 | I7A083 | TGGAACCTA | CAAGCAGAAGACGGCATACGAGATTAGGTTCCAGTCTCGTGGGCTCGG |
| 1 | D11 | I7A084 | CACTTACCA | CAAGCAGAAGACGGCATACGAGATTGGTAAGTGGTCTCGTGGGCTCGG |
| 1 | E11 | I7A085 | ACACAACGC | CAAGCAGAAGACGGCATACGAGATGCGTTGTGTGTCTCGTGGGCTCGG |
| 1 | F11 | I7A086 | TTCTGTTGC | CAAGCAGAAGACGGCATACGAGATGCAACAGAAGTCTCGTGGGCTCGG |
| 1 | G11 | I7A087 | TTCTCAGGA | CAAGCAGAAGACGGCATACGAGATTCCTGAGAAGTCTCGTGGGCTCGG |
| 1 | H11 | I7A088 | CCGTATAAG | CAAGCAGAAGACGGCATACGAGATCTTATACGGGTCTCGTGGGCTCGG |
| 1 | A12 | I7A089 | GGAGCGTAA | CAAGCAGAAGACGGCATACGAGATTTACGCTCCGTCTCGTGGGCTCGG |
| 1 | B12 | I7A090 | TATGGTGGC | CAAGCAGAAGACGGCATACGAGATGCCACCATAGTCTCGTGGGCTCGG |
| 1 | C12 | I7A091 | TTGTTGACC | CAAGCAGAAGACGGCATACGAGATGGTCAACAAGTCTCGTGGGCTCGG |
| 1 | D12 | I7A092 | TTGAACCGG | CAAGCAGAAGACGGCATACGAGATCCGGTTCAAGTCTCGTGGGCTCGG |
| 1 | E12 | I7A093 | CACTGTGGT | CAAGCAGAAGACGGCATACGAGATACCACAGTGGTCTCGTGGGCTCGG |
| 1 | F12 | I7A094 | TTCTCGCTT | CAAGCAGAAGACGGCATACGAGATAAGCGAGAAGTCTCGTGGGCTCGG |
| 1 | G12 | I7A095 | TGGCGTCTT | CAAGCAGAAGACGGCATACGAGATAAGACGCCAGTCTCGTGGGCTCGG |
| 1 | H12 | I7A096 | TCACAACCT | CAAGCAGAAGACGGCATACGAGATAGGTTGTGAGTCTCGTGGGCTCGG |
| 2 | A1 | I7B097 | AGGAGGATC | CAAGCAGAAGACGGCATACGAGATGATCCTCCTGTCTCGTGGGCTCGG |
| 2 | B1 | I7B098 | CCTAACGAA | CAAGCAGAAGACGGCATACGAGATTTCGTTAGGGTCTCGTGGGCTCGG |
| 2 | C1 | I7B099 | TTGGTTGAG | CAAGCAGAAGACGGCATACGAGATCTCAACCAAGTCTCGTGGGCTCGG |
| 2 | D1 | I7B100 | GATAGGAGC | CAAGCAGAAGACGGCATACGAGATGCTCCTATCGTCTCGTGGGCTCGG |
| 2 | E1 | I7B101 | GAGAACGAG | CAAGCAGAAGACGGCATACGAGATCTCGTTCTCGTCTCGTGGGCTCGG |
| 2 | F1 | I7B102 | CCTCAGAAG | CAAGCAGAAGACGGCATACGAGATCTTCTGAGGGTCTCGTGGGCTCGG |
| 2 | G1 | I7B103 | TGGCTTAGT | CAAGCAGAAGACGGCATACGAGATACTAAGCCAGTCTCGTGGGCTCGG |
| 2 | H1 | I7B104 | CGTAATGGA | CAAGCAGAAGACGGCATACGAGATTCCATTACGGTCTCGTGGGCTCGG |
| 2 | A2 | I7B105 | GCTAACAAG | CAAGCAGAAGACGGCATACGAGATCTTGTTAGCGTCTCGTGGGCTCGG |
| 2 | B2 | I7B106 | CCGTGGAAT | CAAGCAGAAGACGGCATACGAGATATTCCACGGGTCTCGTGGGCTCGG |
| 2 | C2 | I7B107 | CAATCGCCA | CAAGCAGAAGACGGCATACGAGATTGGCGATTGGTCTCGTGGGCTCGG |
| 2 | D2 | I7B108 | TTCGCTCCT | CAAGCAGAAGACGGCATACGAGATAGGAGCGAAGTCTCGTGGGCTCGG |
| 2 | E2 | I7B109 | TGCTCTTAC | CAAGCAGAAGACGGCATACGAGATGTAAGAGCAGTCTCGTGGGCTCGG |
| 2 | F2 | I7B110 | AGGCCTTAG | CAAGCAGAAGACGGCATACGAGATCTAAGGCCTGTCTCGTGGGCTCGG |
| 2 | G2 | I7B111 | CCGATGATT | CAAGCAGAAGACGGCATACGAGATAATCATCGGGTCTCGTGGGCTCGG |
| 2 | H2 | I7B112 | GGCCAGAAT | CAAGCAGAAGACGGCATACGAGATATTCTGGCCGTCTCGTGGGCTCGG |
| 2 | A3 | I7B113 | TTGTGTCCG | CAAGCAGAAGACGGCATACGAGATCGGACACAAGTCTCGTGGGCTCGG |
| 2 | B3 | I7B114 | TTACGGTTC | CAAGCAGAAGACGGCATACGAGATGAACCGTAAGTCTCGTGGGCTCGG |
| 2 | C3 | I7B115 | TGTGGCACA | CAAGCAGAAGACGGCATACGAGATTGTGCCACAGTCTCGTGGGCTCGG |
| 2 | D3 | I7B116 | TGGTTACTC | CAAGCAGAAGACGGCATACGAGATGAGTAACCAGTCTCGTGGGCTCGG |
| 2 | E3 | I7B117 | GCAAGCTAA | CAAGCAGAAGACGGCATACGAGATTTAGCTTGCGTCTCGTGGGCTCGG |
| 2 | F3 | I7B118 | TTCCGTGTC | CAAGCAGAAGACGGCATACGAGATGACACGGAAGTCTCGTGGGCTCGG |
| 2 | G3 | I7B119 | TCAGTGGAG | CAAGCAGAAGACGGCATACGAGATCTCCACTGAGTCTCGTGGGCTCGG |
| 2 | H3 | I7B120 | TTCACCAAG | CAAGCAGAAGACGGCATACGAGATCTTGGTGAAGTCTCGTGGGCTCGG |
| 2 | A4 | I7B121 | AGATGTTGC | CAAGCAGAAGACGGCATACGAGATGCAACATCTGTCTCGTGGGCTCGG |
| 2 | B4 | I7B122 | CTGTGTAGA | CAAGCAGAAGACGGCATACGAGATTCTACACAGGTCTCGTGGGCTCGG |
| 2 | C4 | I7B123 | CGCAGACTT | CAAGCAGAAGACGGCATACGAGATAAGTCTGCGGTCTCGTGGGCTCGG |
| 2 | D4 | I7B124 | TGATGTGCT | CAAGCAGAAGACGGCATACGAGATAGCACATCAGTCTCGTGGGCTCGG |
| 2 | E4 | I7B125 | GGACTTGTG | CAAGCAGAAGACGGCATACGAGATCACAAGTCCGTCTCGTGGGCTCGG |
| 2 | F4 | I7B126 | TTCTTGGAC | CAAGCAGAAGACGGCATACGAGATGTCCAAGAAGTCTCGTGGGCTCGG |
| 2 | G4 | I7B127 | CAGACAACA | CAAGCAGAAGACGGCATACGAGATTGTTGTCTGGTCTCGTGGGCTCGG |
| 2 | H4 | I7B128 | AGGAACCAC | CAAGCAGAAGACGGCATACGAGATGTGGTTCCTGTCTCGTGGGCTCGG |
| 2 | A5 | I7B129 | TTCATCGCG | CAAGCAGAAGACGGCATACGAGATCGCGATGAAGTCTCGTGGGCTCGG |
| 2 | B5 | I7B130 | ACCTCCTCA | CAAGCAGAAGACGGCATACGAGATTGAGGAGGTGTCTCGTGGGCTCGG |
| 2 | C5 | I7B131 | TGCGGCTTA | CAAGCAGAAGACGGCATACGAGATTAAGCCGCAGTCTCGTGGGCTCGG |
| 2 | D5 | I7B132 | CAATCCGCT | CAAGCAGAAGACGGCATACGAGATAGCGGATTGGTCTCGTGGGCTCGG |
| 2 | E5 | I7B133 | AACAAGCGG | CAAGCAGAAGACGGCATACGAGATCCGCTTGTTGTCTCGTGGGCTCGG |
| 2 | F5 | I7B134 | GGATCTCAA | CAAGCAGAAGACGGCATACGAGATTTGAGATCCGTCTCGTGGGCTCGG |
| 2 | G5 | I7B135 | CAGCTTCAA | CAAGCAGAAGACGGCATACGAGATTTGAAGCTGGTCTCGTGGGCTCGG |
| 2 | H5 | I7B136 | GGCTAATTC | CAAGCAGAAGACGGCATACGAGATGAATTAGCCGTCTCGTGGGCTCGG |
| 2 | A6 | I7B137 | GGAGACTCA | CAAGCAGAAGACGGCATACGAGATTGAGTCTCCGTCTCGTGGGCTCGG |
| 2 | B6 | I7B138 | TGGCATAAG | CAAGCAGAAGACGGCATACGAGATCTTATGCCAGTCTCGTGGGCTCGG |
| 2 | C6 | I7B139 | ACGCCATTC | CAAGCAGAAGACGGCATACGAGATGAATGGCGTGTCTCGTGGGCTCGG |
| 2 | D6 | I7B140 | AACTGGAAC | CAAGCAGAAGACGGCATACGAGATGTTCCAGTTGTCTCGTGGGCTCGG |
| 2 | E6 | I7B141 | CCAAGGTTG | CAAGCAGAAGACGGCATACGAGATCAACCTTGGGTCTCGTGGGCTCGG |
| 2 | F6 | I7B142 | CAGTTAAGG | CAAGCAGAAGACGGCATACGAGATCCTTAACTGGTCTCGTGGGCTCGG |
| 2 | G6 | I7B143 | CGCCTTAAT | CAAGCAGAAGACGGCATACGAGATATTAAGGCGGTCTCGTGGGCTCGG |
| 2 | H6 | I7B144 | TTCACCTCA | CAAGCAGAAGACGGCATACGAGATTGAGGTGAAGTCTCGTGGGCTCGG |
| 2 | A7 | I7B145 | CCATGTGTG | CAAGCAGAAGACGGCATACGAGATCACACATGGGTCTCGTGGGCTCGG |
| 2 | B7 | I7B146 | CCGCATCAT | CAAGCAGAAGACGGCATACGAGATATGATGCGGGTCTCGTGGGCTCGG |
| 2 | C7 | I7B147 | GGAAGTCCA | CAAGCAGAAGACGGCATACGAGATTGGACTTCCGTCTCGTGGGCTCGG |
| 2 | D7 | I7B148 | TCACTCTAC | CAAGCAGAAGACGGCATACGAGATGTAGAGTGAGTCTCGTGGGCTCGG |
| 2 | E7 | I7B149 | GAGAGTGGA | CAAGCAGAAGACGGCATACGAGATTCCACTCTCGTCTCGTGGGCTCGG |
| 2 | F7 | I7B150 | GCACCTAAC | CAAGCAGAAGACGGCATACGAGATGTTAGGTGCGTCTCGTGGGCTCGG |
| 2 | G7 | I7B151 | TACCACTCT | CAAGCAGAAGACGGCATACGAGATAGAGTGGTAGTCTCGTGGGCTCGG |
| 2 | H7 | I7B152 | TTGACCTTC | CAAGCAGAAGACGGCATACGAGATGAAGGTCAAGTCTCGTGGGCTCGG |
| 2 | A8 | I7B153 | TCACGCCTA | CAAGCAGAAGACGGCATACGAGATTAGGCGTGAGTCTCGTGGGCTCGG |
| 2 | B8 | I7B154 | CATAAGGAG | CAAGCAGAAGACGGCATACGAGATCTCCTTATGGTCTCGTGGGCTCGG |
| 2 | C8 | I7B155 | CGCTCAATT | CAAGCAGAAGACGGCATACGAGATAATTGAGCGGTCTCGTGGGCTCGG |
| 2 | D8 | I7B156 | AAGAGCCGT | CAAGCAGAAGACGGCATACGAGATACGGCTCTTGTCTCGTGGGCTCGG |
| 2 | E8 | I7B157 | CGTAAGAGT | CAAGCAGAAGACGGCATACGAGATACTCTTACGGTCTCGTGGGCTCGG |
| 2 | F8 | I7B158 | CCTTATCCT | CAAGCAGAAGACGGCATACGAGATAGGATAAGGGTCTCGTGGGCTCGG |
| 2 | G8 | I7B159 | AACGGTAGT | CAAGCAGAAGACGGCATACGAGATACTACCGTTGTCTCGTGGGCTCGG |
| 2 | H8 | I7B160 | TTGAGGCCT | CAAGCAGAAGACGGCATACGAGATAGGCCTCAAGTCTCGTGGGCTCGG |
| 2 | A9 | I7B161 | TGGAGGAAT | CAAGCAGAAGACGGCATACGAGATATTCCTCCAGTCTCGTGGGCTCGG |
| 2 | B9 | I7B162 | CTCCATAGA | CAAGCAGAAGACGGCATACGAGATTCTATGGAGGTCTCGTGGGCTCGG |
| 2 | C9 | I7B163 | TATACAGCC | CAAGCAGAAGACGGCATACGAGATGGCTGTATAGTCTCGTGGGCTCGG |
| 2 | D9 | I7B164 | TTCTAGGCG | CAAGCAGAAGACGGCATACGAGATCGCCTAGAAGTCTCGTGGGCTCGG |
| 2 | E9 | I7B165 | TAACCGATC | CAAGCAGAAGACGGCATACGAGATGATCGGTTAGTCTCGTGGGCTCGG |
| 2 | F9 | I7B166 | GAATTGGCC | CAAGCAGAAGACGGCATACGAGATGGCCAATTCGTCTCGTGGGCTCGG |
| 2 | G9 | I7B167 | GAACCTTGT | CAAGCAGAAGACGGCATACGAGATACAAGGTTCGTCTCGTGGGCTCGG |
| 2 | H9 | I7B168 | TTCCGGAGA | CAAGCAGAAGACGGCATACGAGATTCTCCGGAAGTCTCGTGGGCTCGG |
| 2 | A10 | I7B169 | CCACTACAG | CAAGCAGAAGACGGCATACGAGATCTGTAGTGGGTCTCGTGGGCTCGG |
| 2 | B10 | I7B170 | CAATCCAAC | CAAGCAGAAGACGGCATACGAGATGTTGGATTGGTCTCGTGGGCTCGG |
| 2 | C10 | I7B171 | CAAGGTGCT | CAAGCAGAAGACGGCATACGAGATAGCACCTTGGTCTCGTGGGCTCGG |
| 2 | D10 | I7B172 | CGCTTAAGA | CAAGCAGAAGACGGCATACGAGATTCTTAAGCGGTCTCGTGGGCTCGG |
| 2 | E10 | I7B173 | CGATAACCA | CAAGCAGAAGACGGCATACGAGATTGGTTATCGGTCTCGTGGGCTCGG |
| 2 | F10 | I7B174 | TTAGCTTGC | CAAGCAGAAGACGGCATACGAGATGCAAGCTAAGTCTCGTGGGCTCGG |
| 2 | G10 | I7B175 | CCAGTGGTT | CAAGCAGAAGACGGCATACGAGATAACCACTGGGTCTCGTGGGCTCGG |
| 2 | H10 | I7B176 | CCGTCATTA | CAAGCAGAAGACGGCATACGAGATTAATGACGGGTCTCGTGGGCTCGG |
| 2 | A11 | I7B177 | TTACTCACC | CAAGCAGAAGACGGCATACGAGATGGTGAGTAAGTCTCGTGGGCTCGG |
| 2 | B11 | I7B178 | TTCTCTTCG | CAAGCAGAAGACGGCATACGAGATCGAAGAGAAGTCTCGTGGGCTCGG |
| 2 | C11 | I7B179 | TCCGGTTAT | CAAGCAGAAGACGGCATACGAGATATAACCGGAGTCTCGTGGGCTCGG |
| 2 | D11 | I7B180 | TAATGAGCG | CAAGCAGAAGACGGCATACGAGATCGCTCATTAGTCTCGTGGGCTCGG |
| 2 | E11 | I7B181 | CTAGACAGA | CAAGCAGAAGACGGCATACGAGATTCTGTCTAGGTCTCGTGGGCTCGG |
| 2 | F11 | I7B182 | CCATTCCTC | CAAGCAGAAGACGGCATACGAGATGAGGAATGGGTCTCGTGGGCTCGG |
| 2 | G11 | I7B183 | TTGTATGGC | CAAGCAGAAGACGGCATACGAGATGCCATACAAGTCTCGTGGGCTCGG |
| 2 | H11 | I7B184 | CGACAATCC | CAAGCAGAAGACGGCATACGAGATGGATTGTCGGTCTCGTGGGCTCGG |
| 2 | A12 | I7B185 | ACAGAATGG | CAAGCAGAAGACGGCATACGAGATCCATTCTGTGTCTCGTGGGCTCGG |
| 2 | B12 | I7B186 | TAGTTAGGC | CAAGCAGAAGACGGCATACGAGATGCCTAACTAGTCTCGTGGGCTCGG |
| 2 | C12 | I7B187 | TCTGAAGAG | CAAGCAGAAGACGGCATACGAGATCTCTTCAGAGTCTCGTGGGCTCGG |
| 2 | D12 | I7B188 | AAGTAGGTG | CAAGCAGAAGACGGCATACGAGATCACCTACTTGTCTCGTGGGCTCGG |
| 2 | E12 | I7B189 | CAATCTTCC | CAAGCAGAAGACGGCATACGAGATGGAAGATTGGTCTCGTGGGCTCGG |
| 2 | F12 | I7B190 | CAACGATAC | CAAGCAGAAGACGGCATACGAGATGTATCGTTGGTCTCGTGGGCTCGG |
| 2 | G12 | I7B191 | GAAGAGCTA | CAAGCAGAAGACGGCATACGAGATTAGCTCTTCGTCTCGTGGGCTCGG |
| 2 | H12 | I7B192 | TAGGAGTGT | CAAGCAGAAGACGGCATACGAGATACACTCCTAGTCTCGTGGGCTCGG |
| 3 | A1 | I7C193 | TTCTTGTGG | CAAGCAGAAGACGGCATACGAGATCCACAAGAAGTCTCGTGGGCTCGG |
| 3 | B1 | I7C194 | TCCTCAACA | CAAGCAGAAGACGGCATACGAGATTGTTGAGGAGTCTCGTGGGCTCGG |
| 3 | C1 | I7C195 | TGCATCCTT | CAAGCAGAAGACGGCATACGAGATAAGGATGCAGTCTCGTGGGCTCGG |
| 3 | D1 | I7C196 | TTGAGTGTG | CAAGCAGAAGACGGCATACGAGATCACACTCAAGTCTCGTGGGCTCGG |
| 3 | E1 | I7C197 | GGTTAACCT | CAAGCAGAAGACGGCATACGAGATAGGTTAACCGTCTCGTGGGCTCGG |
| 3 | F1 | I7C198 | TGGTTATGG | CAAGCAGAAGACGGCATACGAGATCCATAACCAGTCTCGTGGGCTCGG |
| 3 | G1 | I7C199 | AACCGGCTT | CAAGCAGAAGACGGCATACGAGATAAGCCGGTTGTCTCGTGGGCTCGG |
| 3 | H1 | I7C200 | TGTGGAGCT | CAAGCAGAAGACGGCATACGAGATAGCTCCACAGTCTCGTGGGCTCGG |
| 3 | A2 | I7C201 | TACGGCAAG | CAAGCAGAAGACGGCATACGAGATCTTGCCGTAGTCTCGTGGGCTCGG |
| 3 | B2 | I7C202 | TCGGCCTAA | CAAGCAGAAGACGGCATACGAGATTTAGGCCGAGTCTCGTGGGCTCGG |
| 3 | C2 | I7C203 | TTCCTTGCT | CAAGCAGAAGACGGCATACGAGATAGCAAGGAAGTCTCGTGGGCTCGG |
| 3 | D2 | I7C204 | ACAATAGGC | CAAGCAGAAGACGGCATACGAGATGCCTATTGTGTCTCGTGGGCTCGG |
| 3 | E2 | I7C205 | CAGGACAGT | CAAGCAGAAGACGGCATACGAGATACTGTCCTGGTCTCGTGGGCTCGG |
| 3 | F2 | I7C206 | CGGTGTTAA | CAAGCAGAAGACGGCATACGAGATTTAACACCGGTCTCGTGGGCTCGG |
| 3 | G2 | I7C207 | TTGGATTCC | CAAGCAGAAGACGGCATACGAGATGGAATCCAAGTCTCGTGGGCTCGG |
| 3 | H2 | I7C208 | AAGCCTGGA | CAAGCAGAAGACGGCATACGAGATTCCAGGCTTGTCTCGTGGGCTCGG |
| 3 | A3 | I7C209 | TACCTTCTG | CAAGCAGAAGACGGCATACGAGATCAGAAGGTAGTCTCGTGGGCTCGG |
| 3 | B3 | I7C210 | TTGCATTGG | CAAGCAGAAGACGGCATACGAGATCCAATGCAAGTCTCGTGGGCTCGG |
| 3 | C3 | I7C211 | GGTAAGGTT | CAAGCAGAAGACGGCATACGAGATAACCTTACCGTCTCGTGGGCTCGG |
| 3 | D3 | I7C212 | ACACTCATC | CAAGCAGAAGACGGCATACGAGATGATGAGTGTGTCTCGTGGGCTCGG |
| 3 | E3 | I7C213 | TTCGGTGGT | CAAGCAGAAGACGGCATACGAGATACCACCGAAGTCTCGTGGGCTCGG |
| 3 | F3 | I7C214 | GAGTCAGAG | CAAGCAGAAGACGGCATACGAGATCTCTGACTCGTCTCGTGGGCTCGG |
| 3 | G3 | I7C215 | TCGGCCATT | CAAGCAGAAGACGGCATACGAGATAATGGCCGAGTCTCGTGGGCTCGG |
| 3 | H3 | I7C216 | AAGATGCAG | CAAGCAGAAGACGGCATACGAGATCTGCATCTTGTCTCGTGGGCTCGG |
| 3 | A4 | I7C217 | CCATTGTCC | CAAGCAGAAGACGGCATACGAGATGGACAATGGGTCTCGTGGGCTCGG |
| 3 | B4 | I7C218 | GAATCCGAA | CAAGCAGAAGACGGCATACGAGATTTCGGATTCGTCTCGTGGGCTCGG |
| 3 | C4 | I7C219 | TTCGCCTTG | CAAGCAGAAGACGGCATACGAGATCAAGGCGAAGTCTCGTGGGCTCGG |
| 3 | D4 | I7C220 | TTAGGTGCG | CAAGCAGAAGACGGCATACGAGATCGCACCTAAGTCTCGTGGGCTCGG |
| 3 | E4 | I7C221 | GGCGTAACT | CAAGCAGAAGACGGCATACGAGATAGTTACGCCGTCTCGTGGGCTCGG |
| 3 | F4 | I7C222 | GCCTTCGTT | CAAGCAGAAGACGGCATACGAGATAACGAAGGCGTCTCGTGGGCTCGG |
| 3 | G4 | I7C223 | TAGCGAACA | CAAGCAGAAGACGGCATACGAGATTGTTCGCTAGTCTCGTGGGCTCGG |
| 3 | H4 | I7C224 | CGGAGAATG | CAAGCAGAAGACGGCATACGAGATCATTCTCCGGTCTCGTGGGCTCGG |
| 3 | A5 | I7C225 | TAACACGGA | CAAGCAGAAGACGGCATACGAGATTCCGTGTTAGTCTCGTGGGCTCGG |
| 3 | B5 | I7C226 | TCTGCTGTT | CAAGCAGAAGACGGCATACGAGATAACAGCAGAGTCTCGTGGGCTCGG |
| 3 | C5 | I7C227 | CCGCTTGTA | CAAGCAGAAGACGGCATACGAGATTACAAGCGGGTCTCGTGGGCTCGG |
| 3 | D5 | I7C228 | CAGTTCACC | CAAGCAGAAGACGGCATACGAGATGGTGAACTGGTCTCGTGGGCTCGG |
| 3 | E5 | I7C229 | GAACAGTTG | CAAGCAGAAGACGGCATACGAGATCAACTGTTCGTCTCGTGGGCTCGG |
| 3 | F5 | I7C230 | TGAGTTGGT | CAAGCAGAAGACGGCATACGAGATACCAACTCAGTCTCGTGGGCTCGG |
| 3 | G5 | I7C231 | GGTGATGAT | CAAGCAGAAGACGGCATACGAGATATCATCACCGTCTCGTGGGCTCGG |
| 3 | H5 | I7C232 | TTCCTTAGG | CAAGCAGAAGACGGCATACGAGATCCTAAGGAAGTCTCGTGGGCTCGG |
| 3 | A6 | I7C233 | GGATCAAGG | CAAGCAGAAGACGGCATACGAGATCCTTGATCCGTCTCGTGGGCTCGG |
| 3 | B6 | I7C234 | TCAGTCTCT | CAAGCAGAAGACGGCATACGAGATAGAGACTGAGTCTCGTGGGCTCGG |
| 3 | C6 | I7C235 | CCGTAACAC | CAAGCAGAAGACGGCATACGAGATGTGTTACGGGTCTCGTGGGCTCGG |
| 3 | D6 | I7C236 | TCCACAAGG | CAAGCAGAAGACGGCATACGAGATCCTTGTGGAGTCTCGTGGGCTCGG |
| 3 | E6 | I7C237 | CCGTTCTAA | CAAGCAGAAGACGGCATACGAGATTTAGAACGGGTCTCGTGGGCTCGG |
| 3 | F6 | I7C238 | TTCCTCTGA | CAAGCAGAAGACGGCATACGAGATTCAGAGGAAGTCTCGTGGGCTCGG |
| 3 | G6 | I7C239 | GGCTCTTCT | CAAGCAGAAGACGGCATACGAGATAGAAGAGCCGTCTCGTGGGCTCGG |
| 3 | H6 | I7C240 | TTGGTAAGC | CAAGCAGAAGACGGCATACGAGATGCTTACCAAGTCTCGTGGGCTCGG |
| 3 | A7 | I7C241 | GGATTCGGT | CAAGCAGAAGACGGCATACGAGATACCGAATCCGTCTCGTGGGCTCGG |
| 3 | B7 | I7C242 | CGCCTATCA | CAAGCAGAAGACGGCATACGAGATTGATAGGCGGTCTCGTGGGCTCGG |
| 3 | C7 | I7C243 | ACATCACGG | CAAGCAGAAGACGGCATACGAGATCCGTGATGTGTCTCGTGGGCTCGG |
| 3 | D7 | I7C244 | TTAAGGCTG | CAAGCAGAAGACGGCATACGAGATCAGCCTTAAGTCTCGTGGGCTCGG |
| 3 | E7 | I7C245 | TGCTTGGTG | CAAGCAGAAGACGGCATACGAGATCACCAAGCAGTCTCGTGGGCTCGG |
| 3 | F7 | I7C246 | TTGGCGATA | CAAGCAGAAGACGGCATACGAGATTATCGCCAAGTCTCGTGGGCTCGG |
| 3 | G7 | I7C247 | TCCACAGTT | CAAGCAGAAGACGGCATACGAGATAACTGTGGAGTCTCGTGGGCTCGG |
| 3 | H7 | I7C248 | CAACGCAGA | CAAGCAGAAGACGGCATACGAGATTCTGCGTTGGTCTCGTGGGCTCGG |
| 3 | A8 | I7C249 | TGAGGATTG | CAAGCAGAAGACGGCATACGAGATCAATCCTCAGTCTCGTGGGCTCGG |
| 3 | B8 | I7C250 | TGCGGTATG | CAAGCAGAAGACGGCATACGAGATCATACCGCAGTCTCGTGGGCTCGG |
| 3 | C8 | I7C251 | GGACGCTTA | CAAGCAGAAGACGGCATACGAGATTAAGCGTCCGTCTCGTGGGCTCGG |
| 3 | D8 | I7C252 | TACACTCCG | CAAGCAGAAGACGGCATACGAGATCGGAGTGTAGTCTCGTGGGCTCGG |
| 3 | E8 | I7C253 | CGAACATGA | CAAGCAGAAGACGGCATACGAGATTCATGTTCGGTCTCGTGGGCTCGG |
| 3 | F8 | I7C254 | ACACGTTCC | CAAGCAGAAGACGGCATACGAGATGGAACGTGTGTCTCGTGGGCTCGG |
| 3 | G8 | I7C255 | TTGCGGAAC | CAAGCAGAAGACGGCATACGAGATGTTCCGCAAGTCTCGTGGGCTCGG |
| 3 | H8 | I7C256 | TTATCTCGG | CAAGCAGAAGACGGCATACGAGATCCGAGATAAGTCTCGTGGGCTCGG |
| 3 | A9 | I7C257 | TGACTTCCT | CAAGCAGAAGACGGCATACGAGATAGGAAGTCAGTCTCGTGGGCTCGG |
| 3 | B9 | I7C258 | TTGGCCAAC | CAAGCAGAAGACGGCATACGAGATGTTGGCCAAGTCTCGTGGGCTCGG |
| 3 | C9 | I7C259 | TCCACCATA | CAAGCAGAAGACGGCATACGAGATTATGGTGGAGTCTCGTGGGCTCGG |
| 3 | D9 | I7C260 | CCGCAATAG | CAAGCAGAAGACGGCATACGAGATCTATTGCGGGTCTCGTGGGCTCGG |
| 3 | E9 | I7C261 | CAGGAACTG | CAAGCAGAAGACGGCATACGAGATCAGTTCCTGGTCTCGTGGGCTCGG |
| 3 | F9 | I7C262 | TTCTTGCCA | CAAGCAGAAGACGGCATACGAGATTGGCAAGAAGTCTCGTGGGCTCGG |
| 3 | G9 | I7C263 | AGCGAGGTA | CAAGCAGAAGACGGCATACGAGATTACCTCGCTGTCTCGTGGGCTCGG |
| 3 | H9 | I7C264 | TCCTCCTAT | CAAGCAGAAGACGGCATACGAGATATAGGAGGAGTCTCGTGGGCTCGG |
| 3 | A10 | I7C265 | TTACTGTCG | CAAGCAGAAGACGGCATACGAGATCGACAGTAAGTCTCGTGGGCTCGG |
| 3 | B10 | I7C266 | TAAGCCACA | CAAGCAGAAGACGGCATACGAGATTGTGGCTTAGTCTCGTGGGCTCGG |
| 3 | C10 | I7C267 | CCGACAGAA | CAAGCAGAAGACGGCATACGAGATTTCTGTCGGGTCTCGTGGGCTCGG |
| 3 | D10 | I7C268 | CTAGCTCCA | CAAGCAGAAGACGGCATACGAGATTGGAGCTAGGTCTCGTGGGCTCGG |
| 3 | E10 | I7C269 | GCTCACTAA | CAAGCAGAAGACGGCATACGAGATTTAGTGAGCGTCTCGTGGGCTCGG |
| 3 | F10 | I7C270 | TTGCCTCAT | CAAGCAGAAGACGGCATACGAGATATGAGGCAAGTCTCGTGGGCTCGG |
| 3 | G10 | I7C271 | CAAGGAGAG | CAAGCAGAAGACGGCATACGAGATCTCTCCTTGGTCTCGTGGGCTCGG |
| 3 | H10 | I7C272 | CGAGCTTCT | CAAGCAGAAGACGGCATACGAGATAGAAGCTCGGTCTCGTGGGCTCGG |
| 3 | A11 | I7C273 | TGGTTGTCA | CAAGCAGAAGACGGCATACGAGATTGACAACCAGTCTCGTGGGCTCGG |
| 3 | B11 | I7C274 | CGTACCAAT | CAAGCAGAAGACGGCATACGAGATATTGGTACGGTCTCGTGGGCTCGG |
| 3 | C11 | I7C275 | TAACGCGCT | CAAGCAGAAGACGGCATACGAGATAGCGCGTTAGTCTCGTGGGCTCGG |
| 3 | D11 | I7C276 | TTCTTCCTG | CAAGCAGAAGACGGCATACGAGATCAGGAAGAAGTCTCGTGGGCTCGG |
| 3 | E11 | I7C277 | CCAATATGG | CAAGCAGAAGACGGCATACGAGATCCATATTGGGTCTCGTGGGCTCGG |
| 3 | F11 | I7C278 | AGAACGGCA | CAAGCAGAAGACGGCATACGAGATTGCCGTTCTGTCTCGTGGGCTCGG |
| 3 | G11 | I7C279 | TCCGACCTT | CAAGCAGAAGACGGCATACGAGATAAGGTCGGAGTCTCGTGGGCTCGG |
| 3 | H11 | I7C280 | TTGTCTCTC | CAAGCAGAAGACGGCATACGAGATGAGAGACAAGTCTCGTGGGCTCGG |
| 3 | A12 | I7C281 | AACTAACCG | CAAGCAGAAGACGGCATACGAGATCGGTTAGTTGTCTCGTGGGCTCGG |
| 3 | B12 | I7C282 | TTGTTCCAC | CAAGCAGAAGACGGCATACGAGATGTGGAACAAGTCTCGTGGGCTCGG |
| 3 | C12 | I7C283 | AACGTTGGC | CAAGCAGAAGACGGCATACGAGATGCCAACGTTGTCTCGTGGGCTCGG |
| 3 | D12 | I7C284 | TTGGTGTTC | CAAGCAGAAGACGGCATACGAGATGAACACCAAGTCTCGTGGGCTCGG |
| 3 | E12 | I7C285 | TCATATCCG | CAAGCAGAAGACGGCATACGAGATCGGATATGAGTCTCGTGGGCTCGG |
| 3 | F12 | I7C286 | CGTCTCATA | CAAGCAGAAGACGGCATACGAGATTATGAGACGGTCTCGTGGGCTCGG |
| 3 | G12 | I7C287 | TCGAGGACA | CAAGCAGAAGACGGCATACGAGATTGTCCTCGAGTCTCGTGGGCTCGG |
| 3 | H12 | I7C288 | CACCTGTAT | CAAGCAGAAGACGGCATACGAGATATACAGGTGGTCTCGTGGGCTCGG |
| 4 | A1 | I7D289 | TGTAAGTGG | CAAGCAGAAGACGGCATACGAGATCCACTTACAGTCTCGTGGGCTCGG |
| 4 | B1 | I7D290 | CCGTTGTTG | CAAGCAGAAGACGGCATACGAGATCAACAACGGGTCTCGTGGGCTCGG |
| 4 | C1 | I7D291 | GGCTGGATT | CAAGCAGAAGACGGCATACGAGATAATCCAGCCGTCTCGTGGGCTCGG |
| 4 | D1 | I7D292 | TTGCTATCC | CAAGCAGAAGACGGCATACGAGATGGATAGCAAGTCTCGTGGGCTCGG |
| 4 | E1 | I7D293 | CCGAAGTTC | CAAGCAGAAGACGGCATACGAGATGAACTTCGGGTCTCGTGGGCTCGG |
| 4 | F1 | I7D294 | AACTTCCAC | CAAGCAGAAGACGGCATACGAGATGTGGAAGTTGTCTCGTGGGCTCGG |
| 4 | G1 | I7D295 | GGCAAGACA | CAAGCAGAAGACGGCATACGAGATTGTCTTGCCGTCTCGTGGGCTCGG |
| 4 | H1 | I7D296 | TTCCACTTC | CAAGCAGAAGACGGCATACGAGATGAAGTGGAAGTCTCGTGGGCTCGG |
| 4 | A2 | I7D297 | TTGGACGGA | CAAGCAGAAGACGGCATACGAGATTCCGTCCAAGTCTCGTGGGCTCGG |
| 4 | B2 | I7D298 | TTCGACCAC | CAAGCAGAAGACGGCATACGAGATGTGGTCGAAGTCTCGTGGGCTCGG |
| 4 | C2 | I7D299 | TTCCAAGGC | CAAGCAGAAGACGGCATACGAGATGCCTTGGAAGTCTCGTGGGCTCGG |
| 4 | D2 | I7D300 | TTCTTCTCC | CAAGCAGAAGACGGCATACGAGATGGAGAAGAAGTCTCGTGGGCTCGG |
| 4 | E2 | I7D301 | TTGGTGCAA | CAAGCAGAAGACGGCATACGAGATTTGCACCAAGTCTCGTGGGCTCGG |
| 4 | F2 | I7D302 | CCATCTTGG | CAAGCAGAAGACGGCATACGAGATCCAAGATGGGTCTCGTGGGCTCGG |
| 4 | G2 | I7D303 | TGAGCGGAT | CAAGCAGAAGACGGCATACGAGATATCCGCTCAGTCTCGTGGGCTCGG |
| 4 | H2 | I7D304 | CCATTCAAG | CAAGCAGAAGACGGCATACGAGATCTTGAATGGGTCTCGTGGGCTCGG |
| 4 | A3 | I7D305 | TCCTACCAG | CAAGCAGAAGACGGCATACGAGATCTGGTAGGAGTCTCGTGGGCTCGG |
| 4 | B3 | I7D306 | TTAGGCTCC | CAAGCAGAAGACGGCATACGAGATGGAGCCTAAGTCTCGTGGGCTCGG |
| 4 | C3 | I7D307 | CAACCTACG | CAAGCAGAAGACGGCATACGAGATCGTAGGTTGGTCTCGTGGGCTCGG |
| 4 | D3 | I7D308 | CCACTTATG | CAAGCAGAAGACGGCATACGAGATCATAAGTGGGTCTCGTGGGCTCGG |
| 4 | E3 | I7D309 | CAGCAGATG | CAAGCAGAAGACGGCATACGAGATCATCTGCTGGTCTCGTGGGCTCGG |
| 4 | F3 | I7D310 | TGGTCTTGA | CAAGCAGAAGACGGCATACGAGATTCAAGACCAGTCTCGTGGGCTCGG |
| 4 | G3 | I7D311 | CACATGCAA | CAAGCAGAAGACGGCATACGAGATTTGCATGTGGTCTCGTGGGCTCGG |
| 4 | H3 | I7D312 | TCGTGAGAA | CAAGCAGAAGACGGCATACGAGATTTCTCACGAGTCTCGTGGGCTCGG |
| 4 | A4 | I7D313 | CCTCTATTG | CAAGCAGAAGACGGCATACGAGATCAATAGAGGGTCTCGTGGGCTCGG |
| 4 | B4 | I7D314 | TTGGCAGGT | CAAGCAGAAGACGGCATACGAGATACCTGCCAAGTCTCGTGGGCTCGG |
| 4 | C4 | I7D315 | TGATGGTAG | CAAGCAGAAGACGGCATACGAGATCTACCATCAGTCTCGTGGGCTCGG |
| 4 | D4 | I7D316 | CGGAAGGAA | CAAGCAGAAGACGGCATACGAGATTTCCTTCCGGTCTCGTGGGCTCGG |
| 4 | E4 | I7D317 | TCACTCGTT | CAAGCAGAAGACGGCATACGAGATAACGAGTGAGTCTCGTGGGCTCGG |
| 4 | F4 | I7D318 | CGCAATGTT | CAAGCAGAAGACGGCATACGAGATAACATTGCGGTCTCGTGGGCTCGG |
| 4 | G4 | I7D319 | TTCGAGAGG | CAAGCAGAAGACGGCATACGAGATCCTCTCGAAGTCTCGTGGGCTCGG |
| 4 | H4 | I7D320 | TTCGCCAGA | CAAGCAGAAGACGGCATACGAGATTCTGGCGAAGTCTCGTGGGCTCGG |
| 4 | A5 | I7D321 | TTGTTCAGG | CAAGCAGAAGACGGCATACGAGATCCTGAACAAGTCTCGTGGGCTCGG |
| 4 | B5 | I7D322 | TTCTACCGA | CAAGCAGAAGACGGCATACGAGATTCGGTAGAAGTCTCGTGGGCTCGG |
| 4 | C5 | I7D323 | TACCACAAC | CAAGCAGAAGACGGCATACGAGATGTTGTGGTAGTCTCGTGGGCTCGG |
| 4 | D5 | I7D324 | GGTGACATT | CAAGCAGAAGACGGCATACGAGATAATGTCACCGTCTCGTGGGCTCGG |
| 4 | E5 | I7D325 | CAACTCACT | CAAGCAGAAGACGGCATACGAGATAGTGAGTTGGTCTCGTGGGCTCGG |
| 4 | F5 | I7D326 | TAGAAGCCG | CAAGCAGAAGACGGCATACGAGATCGGCTTCTAGTCTCGTGGGCTCGG |
| 4 | G5 | I7D327 | CGCGGATAA | CAAGCAGAAGACGGCATACGAGATTTATCCGCGGTCTCGTGGGCTCGG |
| 4 | H5 | I7D328 | TTGTCACCT | CAAGCAGAAGACGGCATACGAGATAGGTGACAAGTCTCGTGGGCTCGG |
| 4 | A6 | I7D329 | TGAGGAGAA | CAAGCAGAAGACGGCATACGAGATTTCTCCTCAGTCTCGTGGGCTCGG |
| 4 | B6 | I7D330 | AGAGTTCCG | CAAGCAGAAGACGGCATACGAGATCGGAACTCTGTCTCGTGGGCTCGG |
| 4 | C6 | I7D331 | TTGCGTTCT | CAAGCAGAAGACGGCATACGAGATAGAACGCAAGTCTCGTGGGCTCGG |
| 4 | D6 | I7D332 | TGTAGAGGA | CAAGCAGAAGACGGCATACGAGATTCCTCTACAGTCTCGTGGGCTCGG |
| 4 | E6 | I7D333 | GACTCCTTG | CAAGCAGAAGACGGCATACGAGATCAAGGAGTCGTCTCGTGGGCTCGG |
| 4 | F6 | I7D334 | TGAACATCC | CAAGCAGAAGACGGCATACGAGATGGATGTTCAGTCTCGTGGGCTCGG |
| 4 | G6 | I7D335 | TGGACCTCT | CAAGCAGAAGACGGCATACGAGATAGAGGTCCAGTCTCGTGGGCTCGG |
| 4 | H6 | I7D336 | TCAGGTTGG | CAAGCAGAAGACGGCATACGAGATCCAACCTGAGTCTCGTGGGCTCGG |
| 4 | A7 | I7D337 | TCCTGTCTT | CAAGCAGAAGACGGCATACGAGATAAGACAGGAGTCTCGTGGGCTCGG |
| 4 | B7 | I7D338 | GGATATTCG | CAAGCAGAAGACGGCATACGAGATCGAATATCCGTCTCGTGGGCTCGG |
| 4 | C7 | I7D339 | CGAACTAGG | CAAGCAGAAGACGGCATACGAGATCCTAGTTCGGTCTCGTGGGCTCGG |
| 4 | D7 | I7D340 | TTCTCCACT | CAAGCAGAAGACGGCATACGAGATAGTGGAGAAGTCTCGTGGGCTCGG |
| 4 | E7 | I7D341 | TTACCTGCC | CAAGCAGAAGACGGCATACGAGATGGCAGGTAAGTCTCGTGGGCTCGG |
| 4 | F7 | I7D342 | TTGTGGTGA | CAAGCAGAAGACGGCATACGAGATTCACCACAAGTCTCGTGGGCTCGG |
| 4 | G7 | I7D343 | TTGTGCCTA | CAAGCAGAAGACGGCATACGAGATTAGGCACAAGTCTCGTGGGCTCGG |
| 4 | H7 | I7D344 | CGAGCAATA | CAAGCAGAAGACGGCATACGAGATTATTGCTCGGTCTCGTGGGCTCGG |
| 4 | A8 | I7D345 | TTGGATCTG | CAAGCAGAAGACGGCATACGAGATCAGATCCAAGTCTCGTGGGCTCGG |
| 4 | B8 | I7D346 | TTCGTTCGA | CAAGCAGAAGACGGCATACGAGATTCGAACGAAGTCTCGTGGGCTCGG |
| 4 | C8 | I7D347 | CTGTTGGAA | CAAGCAGAAGACGGCATACGAGATTTCCAACAGGTCTCGTGGGCTCGG |
| 4 | D8 | I7D348 | TTCTTCGGT | CAAGCAGAAGACGGCATACGAGATACCGAAGAAGTCTCGTGGGCTCGG |
| 4 | E8 | I7D349 | TTGAAGGTC | CAAGCAGAAGACGGCATACGAGATGACCTTCAAGTCTCGTGGGCTCGG |
| 4 | F8 | I7D350 | AAGCAGCAT | CAAGCAGAAGACGGCATACGAGATATGCTGCTTGTCTCGTGGGCTCGG |
| 4 | G8 | I7D351 | TAATTCCGG | CAAGCAGAAGACGGCATACGAGATCCGGAATTAGTCTCGTGGGCTCGG |
| 4 | H8 | I7D352 | CTAGTTGTG | CAAGCAGAAGACGGCATACGAGATCACAACTAGGTCTCGTGGGCTCGG |
| 4 | A9 | I7D353 | CATACGCAC | CAAGCAGAAGACGGCATACGAGATGTGCGTATGGTCTCGTGGGCTCGG |
| 4 | B9 | I7D354 | AACCAAGCC | CAAGCAGAAGACGGCATACGAGATGGCTTGGTTGTCTCGTGGGCTCGG |
| 4 | C9 | I7D355 | TCGGTTCTT | CAAGCAGAAGACGGCATACGAGATAAGAACCGAGTCTCGTGGGCTCGG |
| 4 | D9 | I7D356 | CAACACCTT | CAAGCAGAAGACGGCATACGAGATAAGGTGTTGGTCTCGTGGGCTCGG |
| 4 | E9 | I7D357 | TTCTTACGC | CAAGCAGAAGACGGCATACGAGATGCGTAAGAAGTCTCGTGGGCTCGG |
| 4 | F9 | I7D358 | TTGTCGTTG | CAAGCAGAAGACGGCATACGAGATCAACGACAAGTCTCGTGGGCTCGG |
| 4 | G9 | I7D359 | TTAAGGACC | CAAGCAGAAGACGGCATACGAGATGGTCCTTAAGTCTCGTGGGCTCGG |
| 4 | H9 | I7D360 | CCATGTTCT | CAAGCAGAAGACGGCATACGAGATAGAACATGGGTCTCGTGGGCTCGG |
| 4 | A10 | I7D361 | TGCACACGA | CAAGCAGAAGACGGCATACGAGATTCGTGTGCAGTCTCGTGGGCTCGG |
| 4 | B10 | I7D362 | GAACGTGTT | CAAGCAGAAGACGGCATACGAGATAACACGTTCGTCTCGTGGGCTCGG |
| 4 | C10 | I7D363 | TTGCCGGTT | CAAGCAGAAGACGGCATACGAGATAACCGGCAAGTCTCGTGGGCTCGG |
| 4 | D10 | I7D364 | TACCAGGAA | CAAGCAGAAGACGGCATACGAGATTTCCTGGTAGTCTCGTGGGCTCGG |
| 4 | E10 | I7D365 | TTCTCCGTC | CAAGCAGAAGACGGCATACGAGATGACGGAGAAGTCTCGTGGGCTCGG |
| 4 | F10 | I7D366 | GAAGTGACG | CAAGCAGAAGACGGCATACGAGATCGTCACTTCGTCTCGTGGGCTCGG |
| 4 | G10 | I7D367 | TAGGCGGAA | CAAGCAGAAGACGGCATACGAGATTTCCGCCTAGTCTCGTGGGCTCGG |
| 4 | H10 | I7D368 | TTATCGTCC | CAAGCAGAAGACGGCATACGAGATGGACGATAAGTCTCGTGGGCTCGG |
| 4 | A11 | I7D369 | TGATTGCCG | CAAGCAGAAGACGGCATACGAGATCGGCAATCAGTCTCGTGGGCTCGG |
| 4 | B11 | I7D370 | CCAAGAGGA | CAAGCAGAAGACGGCATACGAGATTCCTCTTGGGTCTCGTGGGCTCGG |
| 4 | C11 | I7D371 | GAATGCACC | CAAGCAGAAGACGGCATACGAGATGGTGCATTCGTCTCGTGGGCTCGG |
| 4 | D11 | I7D372 | TTAGTGGCC | CAAGCAGAAGACGGCATACGAGATGGCCACTAAGTCTCGTGGGCTCGG |
| 4 | E11 | I7D373 | AAGTCCTTC | CAAGCAGAAGACGGCATACGAGATGAAGGACTTGTCTCGTGGGCTCGG |
| 4 | F11 | I7D374 | CCACTCGAA | CAAGCAGAAGACGGCATACGAGATTTCGAGTGGGTCTCGTGGGCTCGG |
| 4 | G11 | I7D375 | TTCCATACC | CAAGCAGAAGACGGCATACGAGATGGTATGGAAGTCTCGTGGGCTCGG |
| 4 | H11 | I7D376 | CCAGAAGAC | CAAGCAGAAGACGGCATACGAGATGTCTTCTGGGTCTCGTGGGCTCGG |
| 4 | A12 | I7D377 | TTACCGAGG | CAAGCAGAAGACGGCATACGAGATCCTCGGTAAGTCTCGTGGGCTCGG |
| 4 | B12 | I7D378 | TTCAGGCAA | CAAGCAGAAGACGGCATACGAGATTTGCCTGAAGTCTCGTGGGCTCGG |
| 4 | C12 | I7D379 | CCAACTGGT | CAAGCAGAAGACGGCATACGAGATACCAGTTGGGTCTCGTGGGCTCGG |
| 4 | D12 | I7D380 | CAACCTGTC | CAAGCAGAAGACGGCATACGAGATGACAGGTTGGTCTCGTGGGCTCGG |
| 4 | E12 | I7D381 | TTGGTCGCT | CAAGCAGAAGACGGCATACGAGATAGCGACCAAGTCTCGTGGGCTCGG |
| 4 | F12 | I7D382 | CCGGTACAA | CAAGCAGAAGACGGCATACGAGATTTGTACCGGGTCTCGTGGGCTCGG |
| 4 | G12 | I7D383 | CACACCAGT | CAAGCAGAAGACGGCATACGAGATACTGGTGTGGTCTCGTGGGCTCGG |
| 4 | H12 | I7D384 | AAGTCGTGG | CAAGCAGAAGACGGCATACGAGATCCACGACTTGTCTCGTGGGCTCGG |
