# Supplementary material for: Spider Webs, Soil or Leaf Swabs to Detect Environmental DNA From Terrestrial Vertebrates: What Is the Best Substrate?
Source: Mol Ecol Resour. 2025 Sep 4;25(8):e70037. doi: 10.1111/1755-0998.70037 (PMC12550491; doi:10.1111/1755-0998.70037)
Supplement: Supplementary file 2 — Figure S1: Control sampling site at Montpellier Zoo tropical greenhouse A. Exterior of the tropical greenhouse. B. View of the giant anteater enclosure. C. General view of the greenhouse interior. [file MEN-25-e70037-s002.pdf]

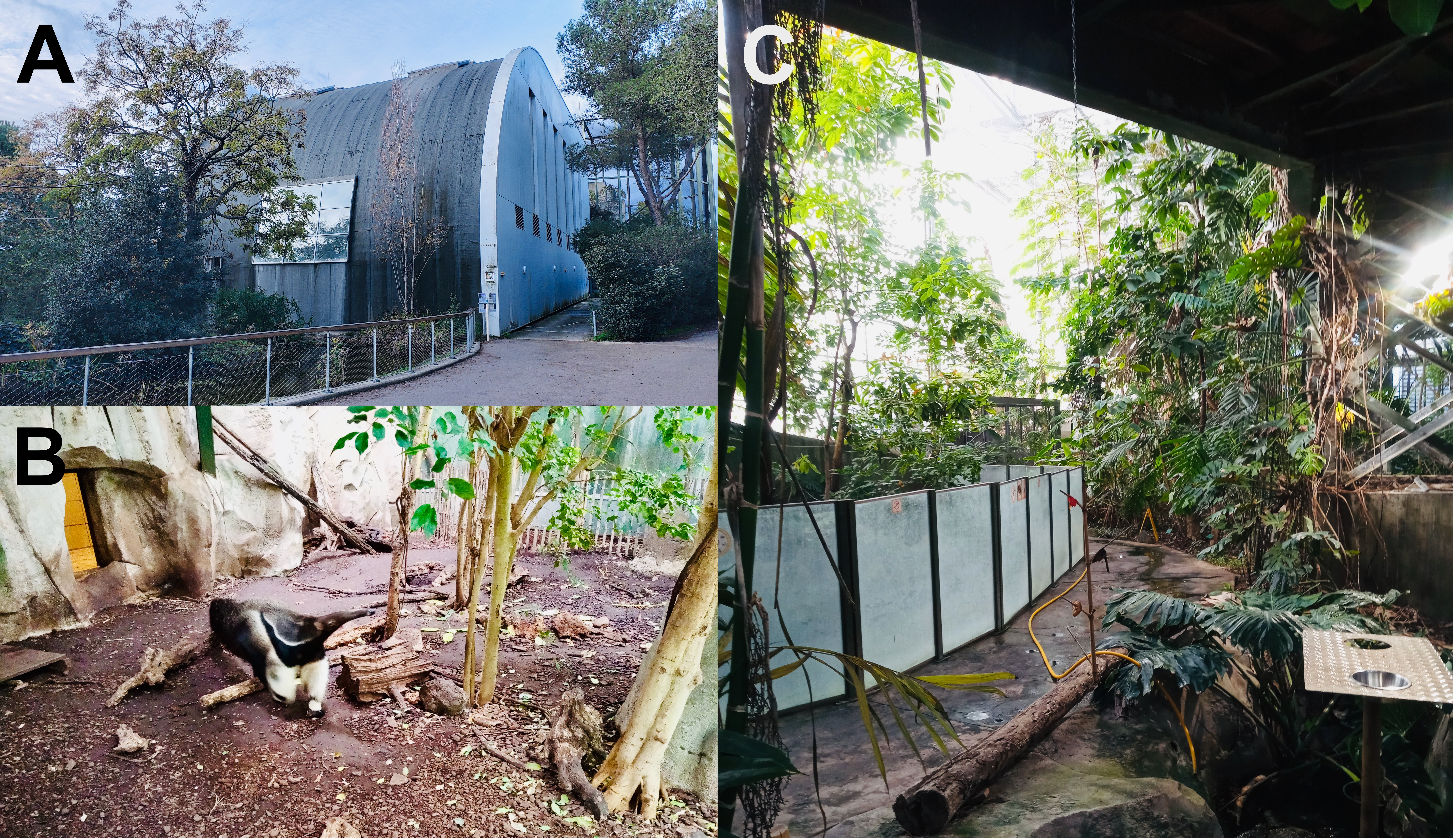

Control sampling site : Montpellier Zoo tropical greenhouse

A. Exterior of the tropical greenhouse. **B.** View of the giant anteater enclosure. **C.** General view of the greenhouse interior.
