# Supplementary material for: Spider Webs, Soil or Leaf Swabs to Detect Environmental DNA From Terrestrial Vertebrates: What Is the Best Substrate?
Source: Mol Ecol Resour. 2025 Sep 4;25(8):e70037. doi: 10.1111/1755-0998.70037 (PMC12550491; doi:10.1111/1755-0998.70037)
Supplement: Supplementary file 3 — Figure S2: Estimated marginal means of within − sample PCR repeatability probability per substrate Bars represent 95% CI. Asterisks indicate significant differences based on a Tukey test (α = 0.05). Substrates include soil, leaf swabs, and spider webs. [file MEN-25-e70037-s003.pdf]

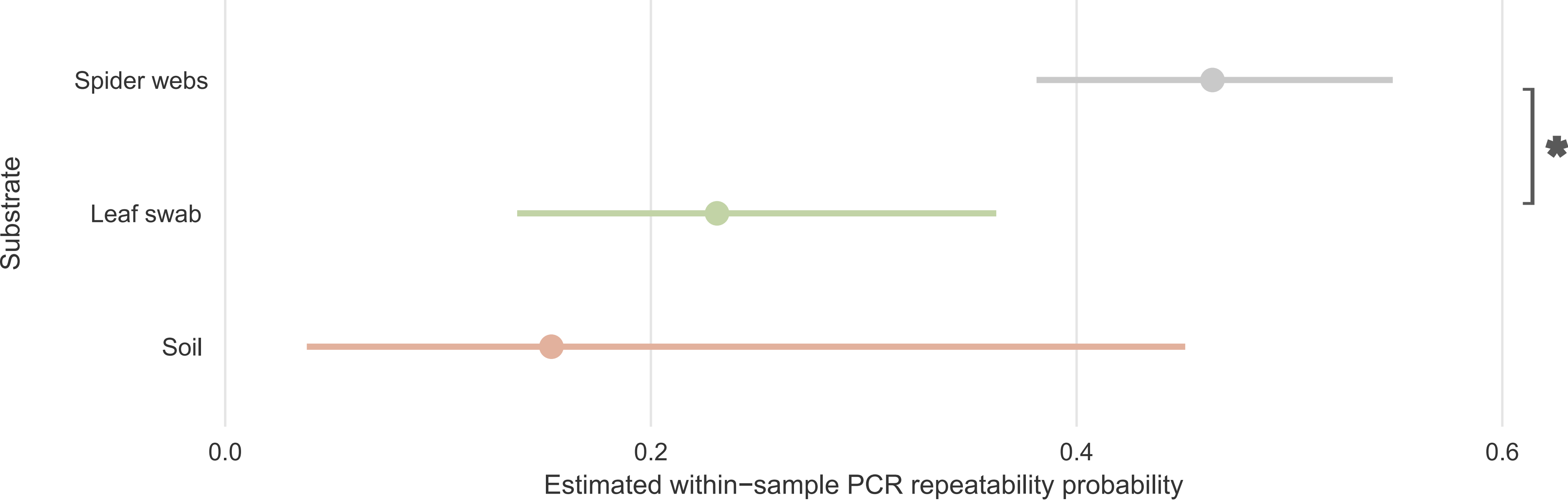

Estimated marginal means of within−sample PCR repeatability probability per substrate

Bars represent 95% IC. Asterisks indicate significant differences based on a Tukey test (α = 0.05).
